# Adaptive Changes in the Vestibular System of Land Snail to a 30-Day Spaceflight and Readaptation on Return to Earth

**DOI:** 10.3389/fncel.2017.00348

**Published:** 2017-11-01

**Authors:** Nikolay Aseyev, Alia Kh. Vinarskaya, Matvey Roshchin, Tatiana A. Korshunova, Aleksey Yu. Malyshev, Alena B. Zuzina, Victor N. Ierusalimsky, Maria S. Lemak, Igor S. Zakharov, Ivan A. Novikov, Peter Kolosov, Ekaterina Chesnokova, Svetlana Volkova, Artem Kasianov, Leonid Uroshlev, Yekaterina Popova, Richard D. Boyle, Pavel M. Balaban

**Affiliations:** ^1^Institute of Higher Nervous Activity and Neurophysiology, Russian Academy of Sciences, Moscow, Russia; ^2^Koltzov Institute of Developmental Biology, Moscow, Russia; ^3^Research Institute of Eye Diseases, Moscow, Russia; ^4^Vavilov Institute of General Genetics, Russia Academy of Sciences, Moscow, Russia; ^5^Space Biosciences Research of NASA Ames Research Center, Moffett Field, CA, United States

**Keywords:** mollusks, gravity sensing, statocyst, neuroplasticity, vestibular system, gravitaxis

## Abstract

The vestibular system receives a permanent influence from gravity and reflexively controls equilibrium. If we assume gravity has remained constant during the species' evolution, will its sensory system adapt to abrupt loss of that force? We address this question in the land snail *Helix lucorum* exposed to 30 days of near weightlessness aboard the Bion-M1 satellite, and studied geotactic behavior of postflight snails, differential gene expressions in statocyst transcriptome, and electrophysiological responses of mechanoreceptors to applied tilts. Each approach revealed plastic changes in the snail's vestibular system assumed in response to spaceflight. Absence of light during the mission also affected statocyst physiology, as revealed by comparison to dark-conditioned control groups. Readaptation to normal tilt responses occurred at ~20 h following return to Earth. Despite the permanence of gravity, the snail responded in a compensatory manner to its loss and readapted once gravity was restored.

## Introduction

Vertebrates and invertebrates sense inertial acceleration and a change in orientation with respect to gravity by mechanoreceptors in the otolith and statolith organs, respectively. The force of gravity is constantly present on Earth and forms the frame of reference for spatial orientation. Inertial accelerations occur within this gravity frame of reference naturally during self-generated movements or those imparted externally onto the organism by perturbations, such as a slippery surface or a deflected leaf. Kreidl (1893) demonstrated in a clever manner that the statocyst is a mechanosensitive organ. As a crayfish molts, it leaves behind the statolith and must manually re-seed its statocyst with a new, typically sand, weight-lending mass. Kreidl replaced the sand with reduced ferrous oxide and manipulated the crayfish's posture using a magnet, thereby simulating the action of the normal gravitational field with a magnetic one. Despite the simplicity of the gastropod organ of balance, there are many functional similarities between the snail's statocyst and the more highly evolved otolith organs of vertebrate species (Popova and Boyle, 2015). Statoreceptors, like hair cells in the vertebrate utricle and saccule, are mechanoreceptors that depend on a weight-lending mass to provide a shearing deformation of the underlying ciliary processes to sense position and acceleration with respect to gravity.

The mechanoreceptors provide the neural code to the reflex mechanisms that control balance and equilibrium and restore the organism to its appropriate posture and orientation. The first vertebrate ear was in essence a graviceptive statocyst (Budelmann, 1988), and only later in evolution did angular acceleration sensation appear as the animal increased its locomotor mobility and developed a flexible neck (Straka et al., 2014).

In vertebrates the vestibular-induced compensatory action is both tonic and dynamic and distributed mainly to the motor circuits controlling the extensor, or antigravity, muscles (Wilson and Jones, 1979). In the absence of gravity and thus an opposing force, even as brief as 5–11 days, astronauts lose about 20% muscle mass (Fitts et al., 2001; Demontis et al., 2017). The changes observed on the skeletomotor system by spaceflight are largely predicted by the mechanical unloading of the body mass: a large skeletal frame to manage muscle contractions about joints is no longer needed and muscle atrophy and osteopenia occur. This same causality might predict a comparable deterioration of vestibular mechanosensory function by space flight: no signal and thus no gain in the system. Surprisingly, despite the permanence of gravity during the evolutionary courses of vertebrates and invertebrates in an otherwise changing environment, the mechanosensory structures do not progressively degenerate, at least not in the short term. Extraordinarily challenging recordings taken during spaceflight from bullfrog otolith afferents by Bracchi et al. (1975) and Gualtierotti (1977) and vestibular neurons and ocular gaze in alert monkeys by Kozlovskaya and her colleagues (reviewed in Cohen et al., 2005) were interpreted as showing an increased sensitivity in measured responses, at least in the early periods of the missions. Otolith afferents in the teleost toadfish exhibited a profound hypersensitivity when tested to inertial accelerations upon return to Earth which recovered back to normal after several days (Boyle et al., 2001). A more recent study in astronauts exposed to long-term space missions aboard the International Space Station have shown a deceased otolith-mediated responses elicited by centrifugation after 6 months of spaceflight which also fully recovered within ~9 days after return (Hallgren et al., 2016). The results in vertebrates exposed to space missions indicate the importance of the duration of exposure to space and the time frame in which measures are taken.

On Earth the orientation of the gravitational acceleration defines the vertical, and although the gravitational vertical is lost in space, the astronaut uses other cues, such as vision to perceive an “up” orientation (Clément et al., 2007). The land snail *Helix lucorum* Linnaeus (*Pulmonata, Gastropoda*) also orients to the vertical using its bilateral statocysts and visual receptors, and can actually move against the force of gravity using a sticky excretion. We selected the land snail for a month-long study on an unmanned orbiting satellite for many reasons, such as their small size permitting a sufficient sample size given our volume limit, snails are hardy and can remain metabolically active under confined conditions for the duration of the mission, snails do not require elaborate life support systems, recordings can be made directly from the sensory receptors themselves (Balaban et al., 2011; Popova and Boyle, 2015), and importantly certain snail behaviors and their underlying cellular circuits are well described (Balaban, 2002). In the present study snails spent 30 days under conditions of weightlessness and darkness, thereby removing its normal cues of the vertical. Under return to Earth the postflight snails were compared to four separate control groups to identify the adaptive changes at the behavioral, structural, molecular, and neurophysiological levels. Based on the present results we also recalculated our previous Foton-M3 data to confirm the findings in separate missions and establish a time course of response readaptation or recovery of function after a spaceflight.

## Materials and methods

### Creating persistent conditions for microgravity

The 2013 Bion-M1 space project was a 30-day orbital research mission operated by the Russian Academy of Sciences, launched on 19th of April (2013) from Baikonur Cosmodrome, Kazakhstan, and landed on 19th of May near Orenburg, Russia. The satellite was pressurized and contained full life support systems to maintain safe temperature, oxygen, and carbon dioxide levels for a wide variety of species. The satellite is under Earth's gravitational pull and is in a state of free fall or microgravity (μG), and thus the objects within the satellite appear to be weightless. Upon return to Earth, the snails were airlifted from the landing site by helicopter to the Orenburg airport, flown by airplane to Moscow, and transported by van to the laboratory, arriving within 13 h after landing.

### Snail populations

Experimental procedures were in compliance with the Guide for the Care and Use of Laboratory Animals published by the National Institutes of Health, and the Ethical Committee of the Institute of Higher Nervous Activity and Neurophysiology of Russian Academy of Sciences approved the protocol. Land snails, *Helix lucorum* L., served as experimental subjects in this study. Snails were collected in vineyards of Crimea, kept in terraria, and fed on cabbage leaves in humid air conditions to prevent aestivation (a prolonged rest in hot and dry periods where the snail covers its shell opening with a special secretion). Experimental animals were assigned to 5 separate groups based on experimental condition (see Table 1). The prime group was the population of postflight snails (PF, *n* = 15) exposed to μG for 30 days on the Bion-M1 orbital satellite. PF snails were housed in the dark during the mission and thus no orientation cues from a light source were available. Four ground-based asynchronous groups were designed for comparison to the PF snails. A Naïve group (NV, *n* = 15) of snails survived for 30 days under normal conditions (Light:Dark cycle was 12:12 and water and food were available *ad libitum*) in a lab terrarium having large spatial dimensions. A Starvation group (ST, *n* = 17) survived for 30 days at the Institute of Biomedical Problems (IMBP, Moscow) facilities as asynchronous control subjects under conditions more closely similar to the PF group, such as a congested habitat, no food or water except a bottom layer of wet paper, a fixed temperature, but a normal light/dark cycle. The third control consisted of snails also mimicking the PF group: a 30-day survival in a flight habitat under comparable conditions, but in the dark (DK group, *n* = 20). Lastly, last group was DK snails exposed to an applied short (2 s) duration 10G acceleration; although this stimulus does not simulate all of the parameters of landing of the Bion-M1 satellite upon its return to Earth, it does present a paradigm of an abrupt transition from a lower gravity level to a higher one. We termed this group a gravity overloading (OL, *n* = 21). Weight-controlled snails of this OL group lived in conditions designed to resemble the PF group except the 30-day exposure to μG. After 30 days of the mock flight the OL snails were centrifuged in individual counterbalanced containers using a Beckman Allegra X-15R (220 RPM, container radius 19.2 cm, 2 s on 10G in manual mode with slowest possible acceleration speed). This procedure neither stopped the crawling behavior of snails nor harmed them in any way by visual observation. Centrifugation of OL snails was performed at the same time of day as the Bion-M1 landing, followed by a 12-h delay of the behavioral experiments, to mimic the conditions and same circadian time as the PF group.

**Table 1:** Sample population of snails.

	**PF**	**OL**	**DK**	**ST**	**NV**
N = (re:weight)	15 (6 + 9)	21 (10 + 11)	20	17 (4 + 13)	15
Snail weight, gm	8–10 (6)	3–7 (11)	18–28	8–10 (4)	20–25
	20–25 (9)	15–20 (10)		20–25 (13)	
Temperature	+	+	+	+	+
Food deprivation and congestion	+	+	+	+	
No light cycle	+	+	+		
Overloading	+	+			
μG exposure	+				

*Five experimental groups: PF, postflight Bion-M1; OL, overload snails subjected to brief hyper-gravity centrifugation before testing; DK, snails housed in flight habitat in dark like PF snails; ST, starvation snails subjected to the same food and water sources and congestion as PF snails; and NV, naïve snails housed in the standard lab terrarium. Sample number (N=) is given for the total number within each group, and where appropriate the snails are partitioned into either a small body weight or large body weight (parentheses). Pluses correspond to the conditions given in the leftmost column in which the snails in each group are subjected*.

### Behavioral studies

Gravitaxis reaction to a 90° head down pitch from horizontal was tested in a previously described apparatus (Balaban et al., 2011) following the same protocol, and full course of this stereotypical behavior was recorded for later analysis using a general purpose digital camera at 30 fps. To provide a double blind control analysis the filenames of the PF, ST, and NV groups were encrypted. Three of the authors (M. R., T. K., and A. Z.) independently split video timelines for each snail to fit the gravitaxis phases as described in the previous report (Balaban et al., 2011). The three analyzers then met, reevaluated their tables of latencies, and made screenshots for every phase of each snail's response. A difference in response latencies of >5 s between the 3 datasets was found in about 3% of measures, thereby triggering a discussion of the merits of each measure until the best measure was determined. The other 2 experimental groups, DK and OL, were investigated roughly a year later and a double blind procedure was deemed not pertinent in this case.

Tentacle withdrawal reaction was measured as the length of each tentacle, first in pixels using ImageJ and then calculated and analyzed as a percentage of full tentacle length (corresponding to T0 phase of gravitaxis response). The angle in degrees between tentacles was measured in ImageJ for every phase of gravitaxis. Accuracy of ImageJ measurements from screenshots was evaluated by mean difference between results of 3 researchers (N. A., A. V., and Y. P.), and the data for 3 randomly chosen animals from PF group and 3 from any of control groups were used. For tentacle length it was 6% error of absolute values, and 4% for inter-tentacle angle measurement. All time parameters were measured with accuracy of 1 s by record timeline on screenshots.

### SEM of inertial mass

Scanning Electron Microscopy (SEM) analysis was made on statocysts from three groups of snails. The statocysts were extracted after the experimental session, frozen dry and stored at −80°C. Before SEM imaging statocysts were fixed by cold absolute ethanol for 12 h, rinsed in distilled water, and placed onto carbon tape in a drop of water. The statocyst was dissected under a dissecting microscope to visualize the inertial mass using fine neurosurgery forceps. After removal of overlying structures, the statoconia preparations were left for few minutes to desiccate. The prepared samples were then placed on carbon tape for SEM and put horizontally on the stage of the microscope (EVO LS10, Carl Zeiss, Germany) for backscattered electrons (BSE) imaging under low vacuum (50–70 Pa) and accelerating voltage of 22 kV (LaB6 cathode). Digital images were captured in tiff format with resolution 3,024 × 2,406 pixels.

### Total RNA preparation and cDNA library construction for NGS

Statocysts of two groups, PF-e and ST (*n* = 4 + 4), were frozen −80°C in microRNA buffer for sequencing. Cellular RNA from statocyst samples was prepared by the guanidine thiocyanate method described by Chomczynski and Sacchi (1987). RNA for cDNA synthesis was treated with DNaseI (Boehringer, Mannheim, Germany) for 30 min at 37°C followed by phenol: chloroform extraction and ethanol precipitation.

Total RNA samples were analyzed using Agilent 2100 Bioanalyzer to confirm RNA isolation purity and absence of RNA degradation. The peak of 28S rRNA is invisible in some species of snails because their 28S rRNA consists of two separate pieces held together by ribosome proteins, and after purification each half of 28S rRNA has the same length as 18S rRNA, so 28S peak merges with 18S peak. According to this, we checked only 18S peak integrity to estimate total RNA quality.

Reverse transcription of RNA samples was performed using oligo-dT(M1) primer and SuperScript® III Enzyme Mix, and second strand of cDNA was created with Mint cDNA synthesis kit (Evrogen). Then we amplified cDNA samples for 27 cycles in the T100 thermal cycler (Bio-Rad) with universal primer M1. Concentration of amplified cDNA was measured using NanoDrop 2000 spectrophotometer. cDNA libraries were then prepared using Ion Plus Fragment library Kit (Ion Torrent, Life Technologies) following manufacturer's protocols. cDNA libraries were purified using Magnetic Bead Cleanup Module (Ion Torrent, Life Technologies).

The quality of each prepared cDNA library was evaluated using Qubit® 2.0 Fluorometer (with Qubit® dsDNA HS Assay Kit) and Agilent 2100 Bioanalyzer (with High Sensitivity DNA chip and Agilent High Sensitivity DNA Kit). In our samples, the amount of short cDNA fragments with length of 25–160 bp did not exceed 10%.

During the next step, clonal amplification was performed using Ion PI™ Template OT2 200 Kit v3 and Ion OneTouch™ 2 system (Life Technologies) in accordance with manufacturer's recommendations. After amplification, the samples were centrifuged, and precipitates were resuspended in bidistilled water and stored at +4°C for 14–18 h.

Sequencing was performed using Ion PI™ Sequencing 200 Kit v3 and Ion PI™ Chip v2 on Ion Proton™ sequencer. One chip contained four libraries tagged with different barcodes.

### Transcriptome analysis of statocysts

First we made *de novo* assembly of *H. lucorum* statocyst transcriptome using the TRINITY v.2.4.0 (32 G memory, 60 threads, min_contig_length = 200), SPAdes v.3.10.0 (-iontorrent flag, 60 threads) and Velvet/Oases (v.1.2.10, optimal k-mer length was estimated by brute-force method and equal 111, -scaffolding and -read_trkg flags was enabled) programs for assembling transcriptome. For assessment programs quality, we have computed for each program N50 values and maximal transcript length (TRINITY N50—321 Maximal Trans. Len.—2,545 bp; SPAdes N50—558 Maximal Trans. Len.—4,813 bp; Oases N50—453 Maximal Trans. Len.—1,262 bp). Next, all assemblies were merged to one FASTA-file. Transcripts from this file were clustered by homology level. Two transcripts have one cluster, if the homology level is more than 90%. The clustering was accomplished by CD-HIT v.4.6 program. Totally from four different assembles were formed 66355 clusters. After clustering, the longest transcripts were extracted from each cluster and wrote into new multi-FASTA file. 1788966 sequences in final assemble were obtained. For each sequence in this assembly was found open reading frame by Transdecoder.

The quality of this combined assembly was estimated by BUSCO v. 2.0.1 program. It computes the percent of correctly assembled orthology genes in the input file. Our combined assembly gave 50% correct orthology genes. For better quality we added an earlier obtained *Helix* neural transcriptome assembly. This “multimerged” assembly gave 95.5% correct orthology genes.

After obtaining the transcriptome reference, we must obtain expression genes level and distinguish important genes. First, we aligned with BWA MEM v. 0.7.5a-r405 each sample to our assembly-reference. Another aligner software (bowtie, bowtie2, tophat) showed worse results (approximately 70% of aligned reads) and wasn't used for further transcriptome analisys. The averaged percent of aligned reads were 93.7% (minimum −91.07%, maximum −95.47% reads). Next, we computed FPKM values for ST group (“control” samples) and PF-e group (“experiment” samples) by CuffDiff software and selected significant differential expressed genes (3546 genes with *p* < 0.05).

### Electrophysiological studies

After the behavioral test was concluded (starting at 14–15 h after landing), the snail was anesthetized by injection of ice-cold isotonic MgCl_2_ solution (~15% of body weight) and its nervous system was isolated and prepared for electrophysiological studies of the statocyst response to tilt mimicking the “head down” and the “tail down” behavioral conditions. The whole nerve recording techniques from the statocyst nerve, *n. vestibularis*, were made during the time window of 15–26 h after landing. The experimental design and data analysis were conducted in a comparable fashion to our previous Foton-M3 study (Balaban et al., 2011). In brief, the central ganglionic ring was dissected free from the anesthetized animal and pinned to a silicone-elastomer (Sylgard)-coated dish. The vestibular nerve was identified, its surrounding connective tissue was removed, and it was placed across a Vaseline bridge that isolated the nerve in one chamber from the nerve, statocyst, and surrounding pedal ganglion in the other chamber. Each chamber contained a saline solution, a Ag/AgCl wire, and the signals from the vestibular nerve were differentially amplified and recorded. The reflex arc from the statocyst nerve to command neurons of the defensive behavior was tested using conventional intracellular recordings in combination with nerve stimulation, single pulse 3 ms width, 0.8–2.1 V, using the isolated stimulation head.

We used the spike sorting by template interactive script provided in Spike2 v.7 (Cambridge Electronic Design, Cambridge, UK) for the single unit analysis. Each record was imported to Spike2 and the cursors selected the key impulse epochs in time with the help of auxiliary channel displaying the movement of the platform. Spike templates were typically made from the first run through the record, and by the second run all the spikes were sorted. First and second principal components were used to visualize clouds of single units. These clouds of single units were checked with PCA to not intersect. Usually default parameters and time window of spike length work well and generate 5–13 additional channels of single unit activity.

### Statistical analysis

Statistical analysis was done in R software using custom written scripts for exploratory statistics and one-way comparison of multiple groups. We performed automated analysis of all data by R Markdown script, which generated statistical reports in doc format, where different statistical approaches were used to the same data. The script, raw data, and statistical reports are uploaded to Open Science Framework platform to make maximal amount of scientific data open (https://osf.io/v5brf/). For the behavioral part of this study we report results of Kruskal-Wallis test (KW) to find the difference between the 5 groups as a default method, because homogeneity of variances assumption mostly fails on our unfiltered data. Dunn's procedure for *post-hoc* pairwise tests against the PF group was used, and the reported *p*-values are after Benjamini-Hochberg adjustment for multiple comparisons (DBH). Alpha 0.05 was accepted as level of significance by default, but exact *p*-values are mostly reported here. Supplementary materials at Open Science Framework contain additional classical statistical inference also as a result of the randomization methods (Bayesian probability and 95% confidence intervals, PERMANOVA) and robust tools (ANOVA on 20% trimmed means and analysis of datasets with Tukey's outliers filtered out), allowing comprehensive analysis of the data. Correlation analysis is provided as Spearman's rank statistics, and correlation matrices were visualized by corrplot R package.

## Results

### Early phases of gravitaxis reaction

**Figure 1:**
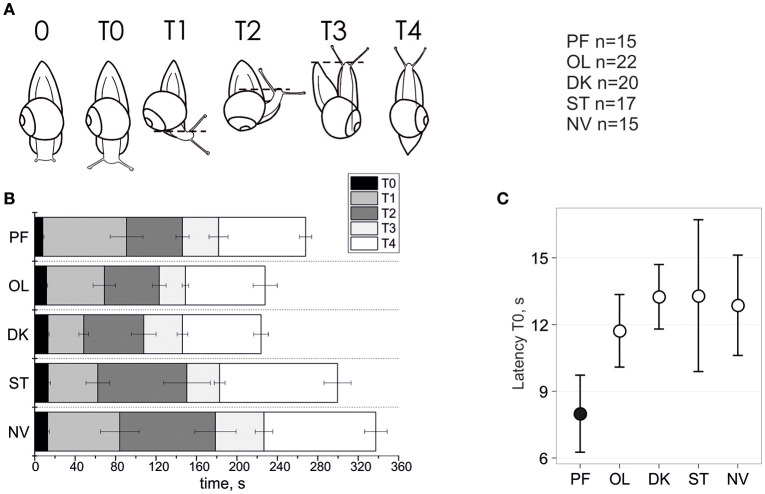
Temporal parameters of snail gravitaxis reaction. **(A)** Designation of 6 gravitaxis phases based on stereotypic behavior of snails. **(B)** Start, stop, and duration of the 6 gravitaxis phases (in sec) in the 5 separate snail groups (bars, ± Standard Error of the Mean). **(C)** Latency of T0 phase (tentacles elongation) in all snail groups, mean and 95% CI.

**Figure 2:**
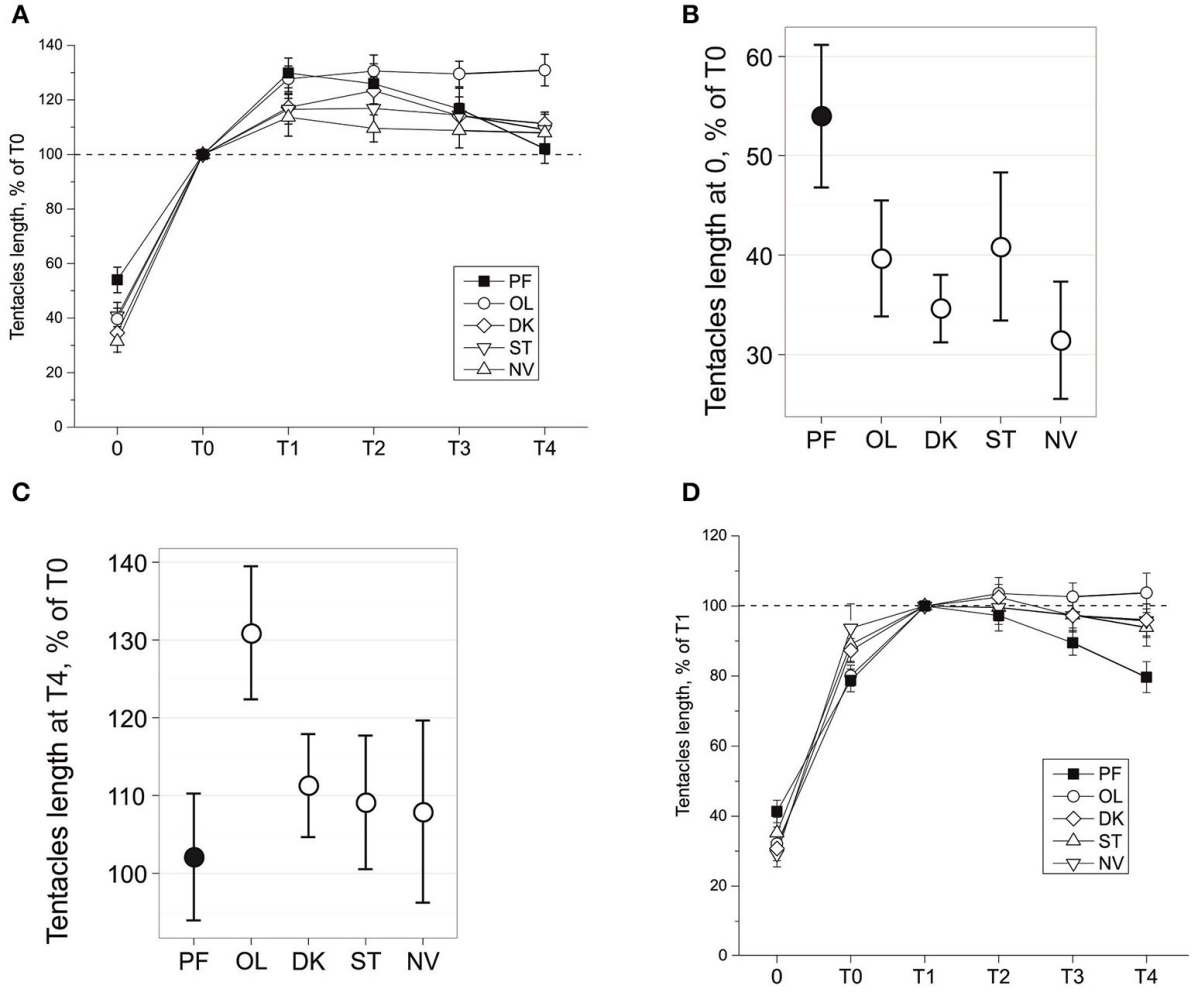
Tentacles length during snail gravitaxis. **(A)** Tentacles length during all gravitaxis phases, normalized for *T0* = 100%. Tentacles length in 0 **(B)** and T4 **(C)** gravitaxis phases, mean and 95% CI. **(D)** Tentacles length during all gravitaxis phases, normalized for *T1* = 100%.

When the platform upon which the snail is attached is suddenly pitched from horizontal to vertical, the snail displays a fear-like reaction by fully or partially withdrawing its tentacles (designed phase 0; Figure 1A). This reaction (latency of withdrawal is <1 s) is believed mediated by the gravi-sensing statocyst organ, the snail's equivalent to a vertebrate's vestibular otolith organ. Therefore, the differences in magnitude and time (duration) of the resulting tentacle elongation phase likely reveal changes in reaction of the snail's statocyst. We define the phase of fast elongation of tentacles before the snail starts to turn its body as the T0 phase of gravitaxis (Figure 1A). Our previous report (Balaban et al., 2011) did not follow this convention, and thus the behavior seen in phase T0 of Figure 2A does not correctly align with our current convention because the length of tentacles were not fully extended. At the end of T0 phase the rapid tentacle elongation is complete and the black eyes on their tips are clearly recognizable in most of the records, both of which occur before the head actually starts to move. Snails often start turning to T1 phase immediately after some amount of “hesitation” or scanning the space around them by moving the head from side to side. There was no significant difference between groups in the count of scanning movements (*p* = 0.114 by Fisher's Exact Test with Monte-Carlo simulated *p*-value based on 99999 replicates); 82 of 88 (93%) snails of all groups turned the body to position the head above the shell in T2 phase.

### Temporal parameters (latencies and durations) of gravitaxis phases

The dynamics of gravitaxis behavior are shown in Figure 1. A comparison of phase T0 duration between the postflight (PF) to the four control groups reveals statistically significant shortening (panel C) of T0 in PF snails (KW *p* < 0.006, DBH *p*_*adj max*_ = 0.011 for PF-OL comparison; mean ± 95% CI). Due to the nature of this measured parameter, there are few extreme outliers in the dataset (*q.v*. Supplementary Materials). Robust methods are recommended to deal with such samples to improve the power of statistics, and indeed they return *p* ≤ 0.001 in KW and DBH tests, thereby providing strong support for a shorter phase T0 in PF than in the other four control groups of snails. No statistical differences of T0 latency were found between the four control groups, even with the application of powerful randomization methods on filtered datasets.

A comparison of phase T1 latency between PF to the four control groups shows no statistically significant difference between them (KW *p* = 0.195). There are few outliers in the dataset, so we applied robust statistics as well, which resulted only in marginal significance (KW *p* = 0.039, DBH *p*_*adj*_ = 0.01 for PF-ST only). A similar result was found with T1 duration (T1 latency–T0 latency), with an insignificant difference between groups (KW *p* = 0.106) in unfiltered dataset and significant for only two control groups if Tukey's outliers were filtered out (KW *p* = 0.02, DBH *p*_*adj*_ = 0.003 for PF-ST and *p*_*adj*_ = 0.023 for PF-DK).

With respect to phase T2 latency a similar result was observed. The comparison of this phase between PF to the four control groups again showed no statistically significant difference between them (KW *p* = 0.223). There are a few outliers in the dataset, so we used robust statistics here as well, which resulted in only marginal significance (KW *p* = 0.046, DBH reveals no significant *p*_*adj*_-values). No significant differences for T2 duration, and also for the later phases T3 and T4 latency and duration, were found or at best a marginal one between two groups in comparison only (*q.v*. Supplementary Materials for details).

### Tentacles withdrawal during gravitaxis reaction

Changes of tentacle length during the gravitaxis behavior are shown in Figure 2A. No significant asymmetry in tentacle withdrawal reaction was found in any group (*p* = 0.483, dependent *t*-test), and thus data for left and right tentacles were averaged. A comparison of phase 0 tentacle withdrawal reaction (Figure 2B) of the four control groups to the PF group reveals that PF snails are retracting their tentacles to a lesser degree than the snails in each control group (KW *p* = 0.009, DBH *p*_*adj*_ < 0.035 for 3 of 4 control groups, and insignificant *p*_*adj*_ = 0.1 for PF-ST comparison).

The later dynamics of the tentacle lengths show more complicated differences between the groups. After a relatively fast elongation of the tentacles during phase T0 (8–15 s), the snails in all groups slowly elongated their tentacles ~10–30% more during T1 phase (Figure 2A). After phase T1 tentacle length reached a plateau in all control groups, with a higher length seen in OL snails than in the NV, ST, and DK snails (insignificantly during T1-T2, at tendency level (*p* < 0.1) at T3, and significantly for T4 (against all groups including PF: KW *p* = 0.02, DBH *p*_*adj max*_ = 0.049; Figure 2C). The tentacle length of PF snails during the late gravitaxis phases showed different dynamics (Figure 2A). At T1 it reached a maximum (~130% of T0, close to OL group value), and then gradually declined over time to ~T0 length at the end of T4 phase. The same data in Figure 2A are normalized in Figure 2D for tentacle length at phase T1 as 100%, but the procedure did not change the direction of observed effects.

### Inter-tentacle angle during gravitaxis reaction

The dynamics of the angle between tentacles was measured in certain phases of gravitaxis reaction and are shown in Figure S1A. No significant differences between groups were found except the delta angle (T0-0) difference, which is significant for PF-NV and OL-NV comparisons (KW *p* = 0.013, DBH *p*_*adj*_ = 0.029 and *p*_*adj*_ = 0.019; Figure S1B). More powerful statistical methods were applied but failed to reveal any additional differences among the snail groups (*q. v*. Supplementary Materials).

### Correlation analysis of gravitaxis reaction

**Figure 3:**
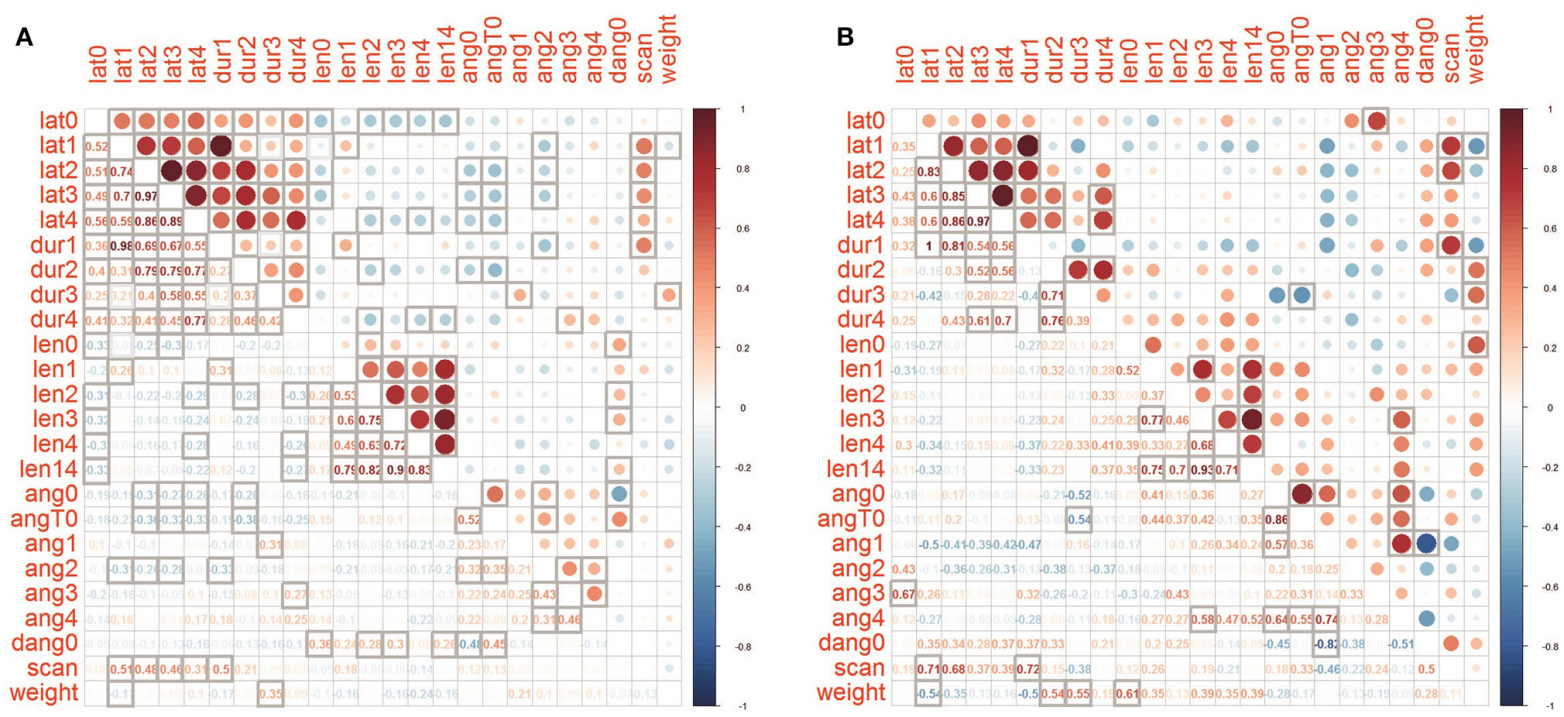
Correlation matrices of parameters for **(A)** joined controls and **(B)** PF group. Cells with bold borders are significant (*p* < 0.05), below main diagonal the Spearman's rho values are shown.

A correlation analysis was made between the merged datasets of the four control groups and the PF dataset (Figure 3). Phase T1-T4 latencies and durations are positively correlated among themselves in both PF and joined control group of snails, as well as tentacle length, and, to a lesser amount, the inter-tentacle angles. In the PF group correlations between inter-tentacle angles of early and late phases is higher than in controls. Delta (T0-0) inter-tentacle angle is weakly positively correlated with tentacle length in controls but not in the PF group. In PF group delta (T0-0) is correlated with latency, but no such a correlation was seen in the merged control. As expected, the number of head scanning movements is positively correlated with latency for all phases. In control dataset tentacle lengths show weak negative correlations with temporal parameters (most pronounced and significant for T0 latency and length, Figure 3A). For the PF group no such a correlation was found (Figure 3B). Latencies and inter-tentacle angles show weak negative correlations in both PF and control groups. In PF group tentacle lengths show weak positive correlations with inter-tentacle angle (although insignificant with the sample size used), in contrast to control dataset showing no such correlation. Positive correlation of snail weight with duration of “ascending” phases T2 and T3 is more pronounced in PF group then in control, also as negative correlation with duration of T1.

### Foton-M3 behavioral data reanalysis

The behavioral data from a 2007 Foton-M3 12-day orbital satellite mission were analyzed before for latency of gravitaxis phases (Balaban et al., 2011), but the small sample size did not allow us to make any statistically supported conclusions. The design of Foton-M3 experiment was slightly different: gravitaxis responses only up to phase T2 were recorded, and the same preflight snails were used as control group. Here we reanalyze the Foton-M3 behavioral records following the same workflow as Bion-M1 data to test the confidence of our main findings (Figure S2). The latency of T0 phase of Foton-M3 postflight group had a tendency to be shorter than in their preflight control measures (median 9.5 vs. 12.5 s, *n* = 10, *p* = 0.112 in dependent *t*-test). Tentacle withdrawal reaction in postflight group of snails was weaker than in the same snails in preflight tests (median 60.45 vs. 34% of T0 length, *p* = 0.002 in dependent *t*-test), and showed no difference with the control in late phases (Figure S2B). Differences in the inter-tentacle angles in Foton-M3 data (Figure S2C) are close to significance level: for postflight group phase 0 and T0 angles were smaller, than for preflight control (*p* = 0.025 and *p* = 0.067 correspondingly in dependent *t*-test); the delta (T0-0) angle was higher in postflight group (*p* = 0.051 in dependent *t*-test). Conclusively, Foton-M3 data reanalysis revealed the differences in postflight 2007 group in the same direction as in Bion-M1 postflight group.

### Statoconia structure

**Figure 4:**
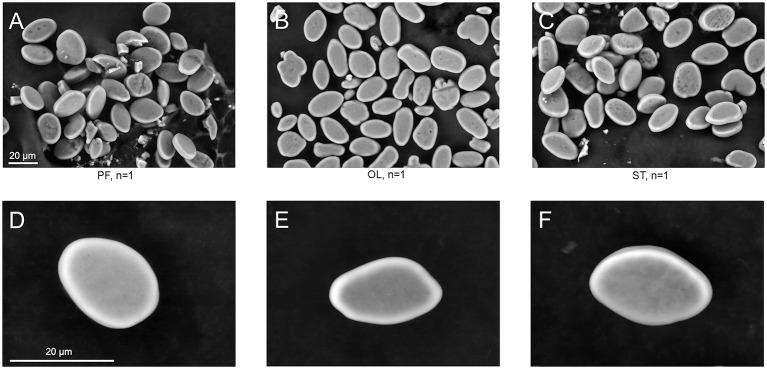
SEM images of statoconia. **(A–C)**, Low magnification view used for area calculations; **(D–F)**, Maximal magnification of typical statoconia. Columns represent statoconia preparations from different groups: **(A,D)**, PF group; **(B,E)**, OL group; **(C,F)**, ST group.

The surface structure of the statoconia mass from groups PF, OL and ST was imaged by SEM to examine the role of possible statoconia growth after 30-day exposure to μG conditions (Figure 4). Although the sample size is small (one statocyst in each group), no crustations or “flake-like” structures were observed in the statoconia of the PF sample that might indicate a deposition of calcium carbonate; no differences were found in either statoconia shape or surface structure between groups. The statoconia areas were calculated from SEM images in ImageJ ROI manager, and a tracing tool with minimal manual correction selected each region. No significant difference was found between groups (μm^2^, PF: 218.9 ± 12.24, OL: 196.6 ± 12.59, ST: 235.5 ± 14.8, n. s. *p*_*adj*_ ≥ 0.43 in DBH pairwise comparisons against PF group).

### Transcriptome analysis of statocysts

**Figure 5:**
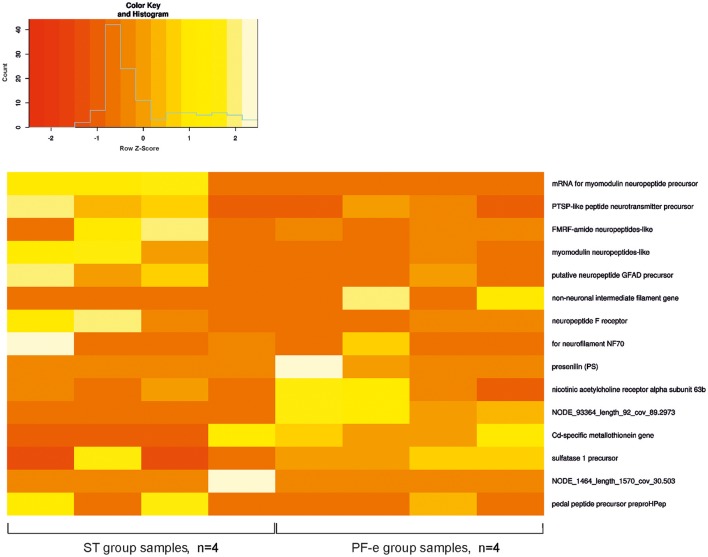
The heatmap for FPKM value of most significant differential expression genes.

We allocated 3,544 transcripts with a significant difference between FPKM values in the control (ST) and experiment (PF-e) groups. These transcripts were annotated by BLAST homology finding. NP database was used for alignment. There were 2,288 proteins annotated. It is useful to note that many transcripts have similar annotation. For example, Cd-specific metallothionein gene was found 373 times. Possible explanations may be a clustering of similar genes or a very aggressive PCR. Fifty of the most significant differential expression genes (*p* < 0.05) are listed in Table S1 and the most interesting of them are shown on the heatmap diagram of Figure 5. Among the most significant differentially expressed genes are the neuronal specific precursors of neuropeptides, neurotransmitter receptor subunits, and other genes involved in cell signaling. This result of transcriptome analysis unambiguously identified the statocyst itself as the site of neural plasticity during the process of readaptation to normal gravity after spaceflight. Raw data were deposited at NCBI Bioproject, accession number PRJNA400816.

### Electrophysiological study of statocyst reflex arc

**Figure 6:**
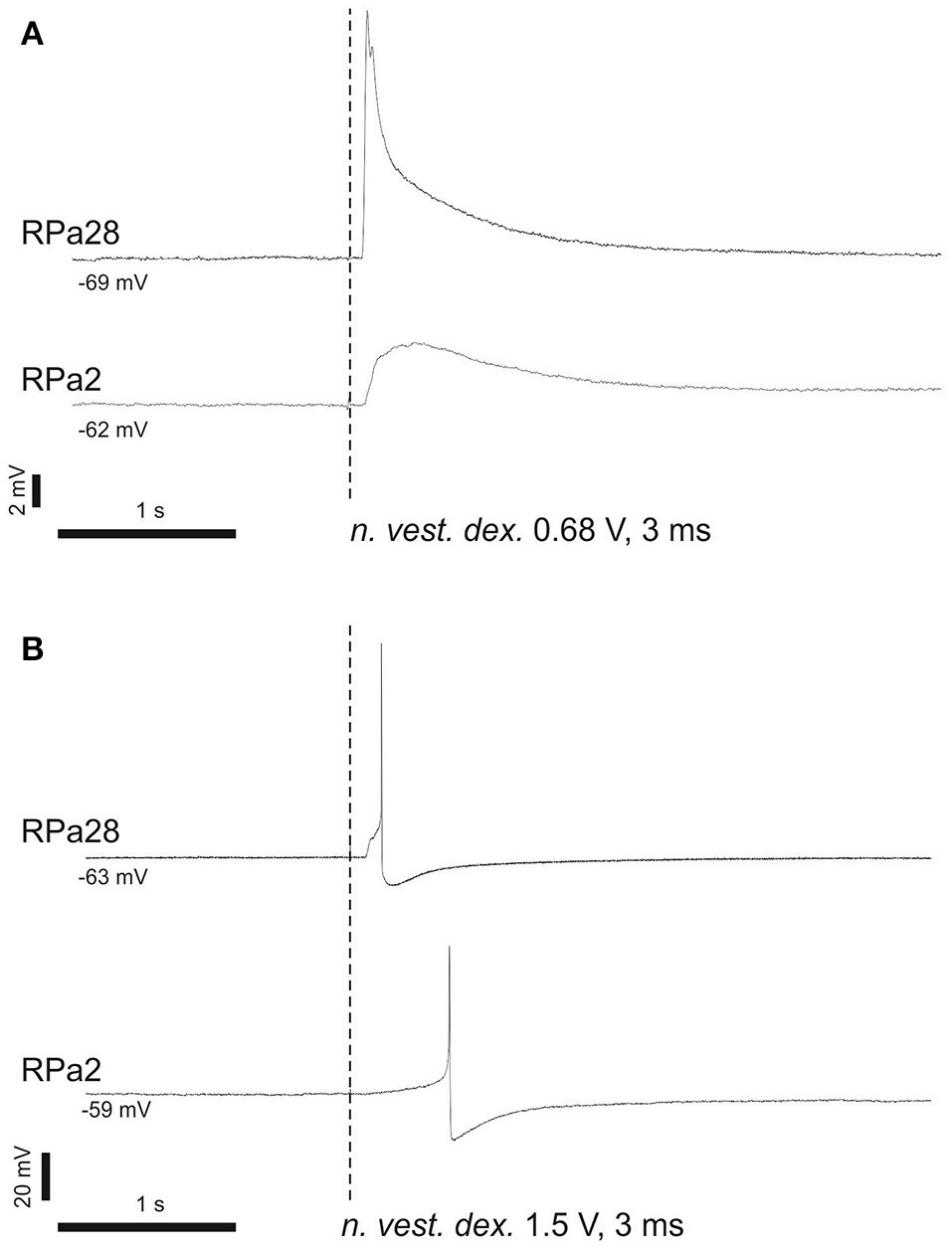
Command neurons of defensive behavior receive excitatory input from vestibular nerve. **(A)** Single shock 3 ms, 0.68 V; **(B)** single shock 3 ms, 1.5 V. Stimulation of *n. vestibularis* exerts EPSP **(A)** or an action potential **(B)** in ipsi- and contralateral command neurons for defensive behavior.

In naïve snails we studied the reflex arc from statocyst sensory neurons to command and motoneurons for a defensive withdrawal behavior. Brief and weak electric shocks, delivered to statocyst nerve, exert an excitatory postsynaptic potential (EPSP) or triggers an action potential in Pa2 and Pa28 neurons (Figure 6). The EPSP shape and its latency suggest a polysynaptic mechanism via unidentified interneurons.

### Background statocyst activity

In Bion-M1 postflight and the four control groups we recorded background statocyst activity and induced responses to tilts in different directions. Background firing rate in preparations of different groups was not statistically different, although OL and especially DK preparations had increased level of background activity (spikes per second, PF: 6.46 ± 0.7, OL: 8.14 ± 0.89, DK: 10.25 ± 1.0, ST: 6.6 ± 0.55, NV: 6.94 ± 1.1; DBH all against PF n. s., minimal *p*_*adj*_ = 0.072 for PF-DK comparison, more statistics are available as Supplementary Materials).

### Electrophysiological studies: technical controls

Many factors can affect the recorded responses of statocysts, and thus we attempted to control as many of them as possible to reveal the effect of microgravity exposure on the vestibular system. Short-term plasticity (habituation or sensitization) during an experiment can affect neural responses, so our protocol controlled it by a fixed inter-stimulus interval and a fixed sequence of stimuli, where the first four tilts were excluded from the analysis. Additional experiments were made to study the stability of nerve responses to a long sequence of stimuli (exhaustive control, Figure S3), and to a sequence with a reversed order of stimuli (Figure S4). There is also the possibility of long-term circadian changes of responses, as it was shown for the snail lip sensory nerve (Voss et al., 1997); this factor might influence the results because the recordings of PF snails commenced after their arrival to the lab at night. To exclude such a possibility we provide additional experiments with our isolated CNS-statocysts preparations (Figure S5). Results of these technical controls allow us to analyze the data acquired by the main protocol.

### Postflight increase of statocyst response to vestibular stimulation

**Figure 7:**
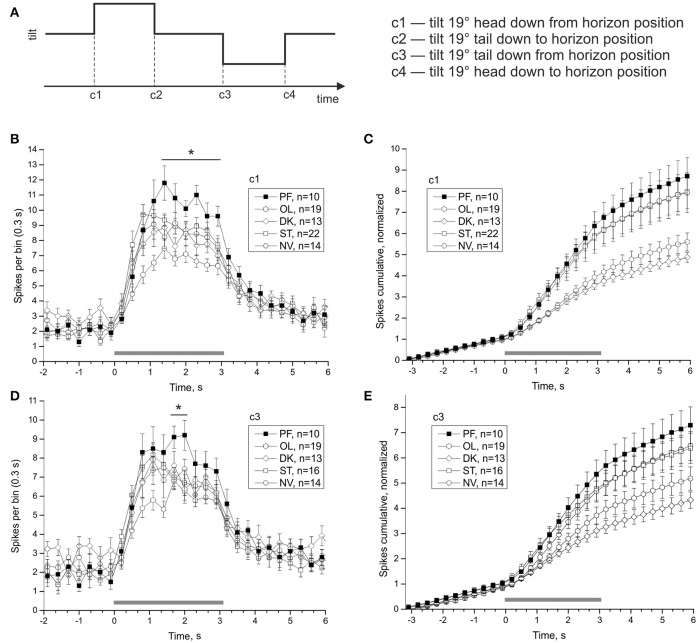
Averaged statocysts responses to vestibular stimuli. **(A)** Sequence of presented tilts. Upward displacement of the platform corresponds to head-down tilt of the animal tilt. **(B,D)**, Peri-stimulus curves of averaged responses to stimuli in two opposite directions. **(C,E)**, Cumulative curves of responses for the same stimuli, normalized to background firing rate. ^*^p < 0.05.

Averaged statocyst responses to stimuli for all groups are shown in Figure 7. The number of spikes in the range (1.4–3.2) s in response to head-down tilt (c1) is significantly greater in PF snails than that in other snails (PF: 80.4 ± 4.44, OL: 52.58 ± 3.42, DK: 61.85 ± 4.55, ST: 68.68 ± 3.26, NV: 65.21 ± 5.05; DBH all against PF *p*_*adj*_ ≤ 0.026 after removing of 3 outliers in ST group). The number of spikes in the range (1.7–2.0) s in response to tilt in direction tail down (c3) is significantly more in group PF than in other groups (PF: 18.3 ± 1.24, OL: 13.05 ± 0.83, DK: 14.31 ± 1.47, ST: 13.88 ± 0.96, NV: 14.29 ± 0.87; DBH all against PF *p*_*adj*_ ≤ 0.045). Both adjusted *p*-values are close to marginal value, so these results must be viewed with caution. Control groups show no homogeneity: preparations of two groups with more controlled factors, OL and DK, responded with smaller gain (number of spikes after the tilt, normalized to background firing rate) than preparations of NV and ST groups, Figures 7C,E (for c1 OL: 3.93 ± 0.3, DK: 3.56 ± 0.21, ST: 5.93 ± 0.6 NV: 5.82 ± 0.75, DBH *p*_*adj*_ ≤ 0.007).

### Adaptation to microgravity

**Figure 8:**
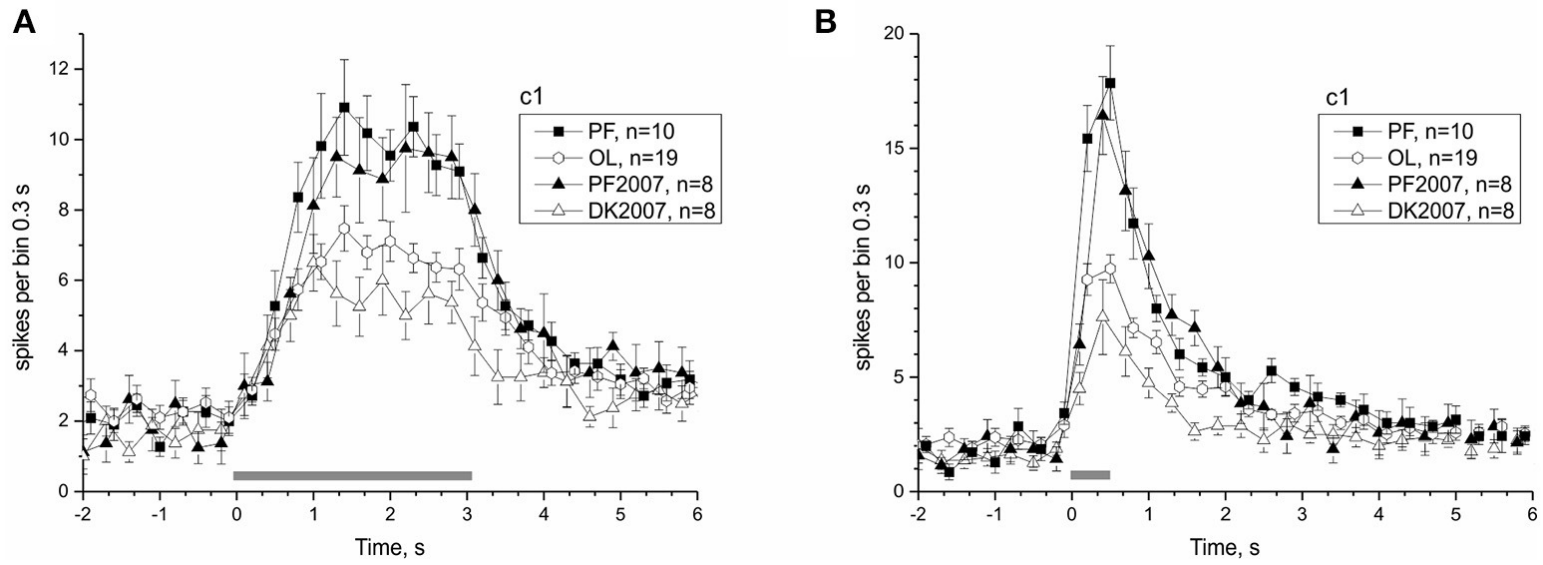
Comparison of Bion-M1 and Foton-M3 postflight groups reveals an independence of microgravity adaptation on mission duration. **(A)** Response to c1 stimulus with duration 3,050 ms. **(B)** Response to c1 stimulus with duration 550 ms. Filled symbols represent PF snails and open symbols the corresponding control snails (see legend box).

The 30-day exposure to μG conditions in the Bion-M1 mission, as well as the shorter 12-day duration of the Foton-M3 mission, significantly affected the responses of the PF snails. Postflight snails of both projects arrived at the lab at comparable times (first nerve recording ~14 h after landing), and the same protocol was used in all preparations. As a result we can compare neural responses to identical tilt stimuli in the two postflight groups to check the dependence of statocyst adaptation to μG on the duration of mission. Figure 8 shows the comparison of statocyst tilt responses to mission duration. No statistically significant difference in number of spikes between the two postflight groups was found in pairwise comparisons [to tilt 3,050 ms range (1.1–2.9) s PF: 70.15 ± 3.84 vs. PF2007: 72.5 ± 8.042, KW *p* = 0.72; for tilt 550 ms range (0.2–1.4) s PF: 52.59 ± 2.73 vs. PF2007: 54 ± 5.74, KW *p* = 0.8], suggesting the response adaptation to μG reaches a plateau at a time shorter than 12 days.

### Readaptation to normal gravity

**Figure 9:**
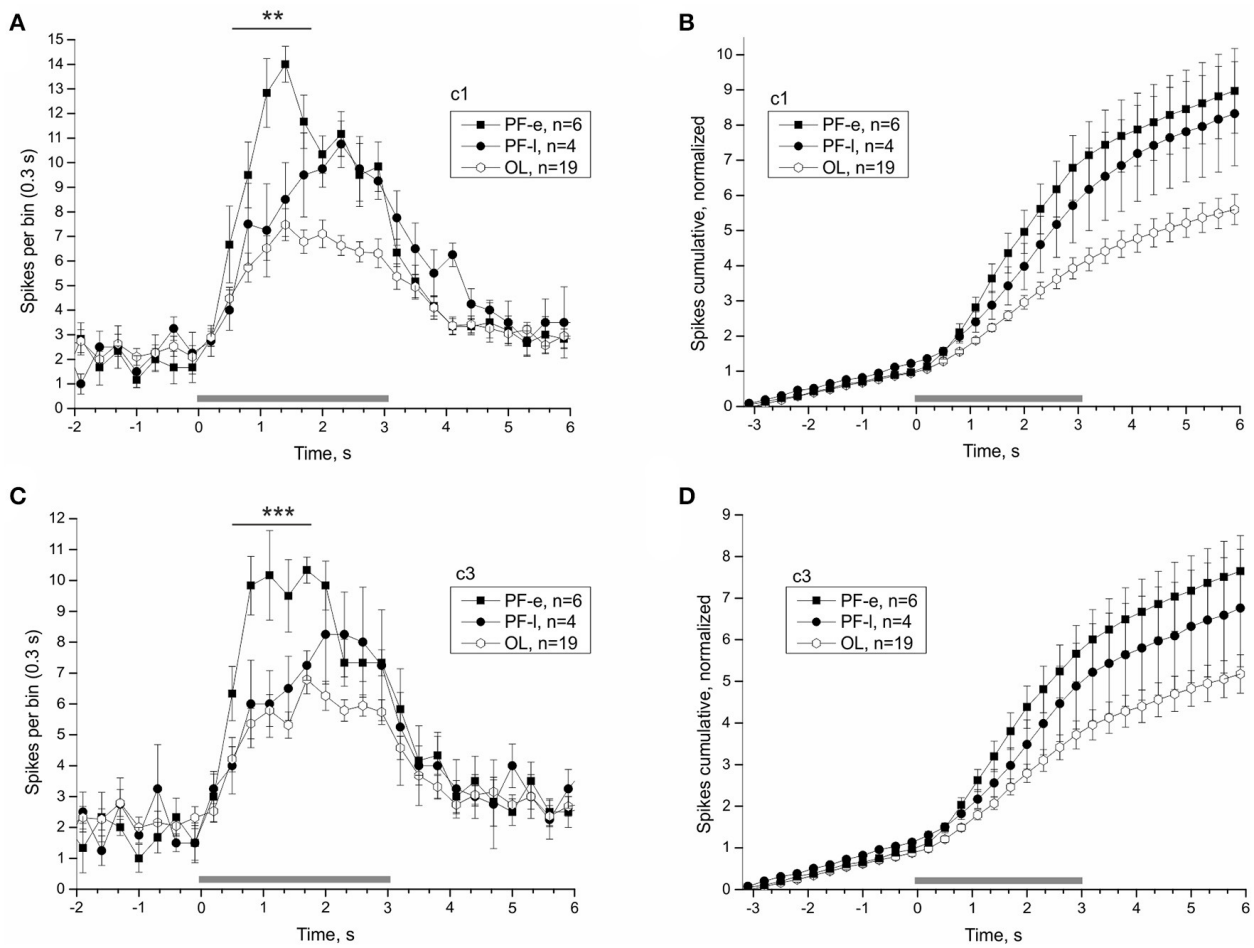
Splitting PF group by the time of recordings after touchdown reveals the time course of the re-adaptation process. Averaged statocysts responses to vestibular stimuli of the early (PF-e) and late (PF-l) together with the overload (OL) control snails are given in each plot. The PF-e subgroup shows the largest magnitude of response. **(A,C)**, Peri-stimulus curves of averaged responses to stimuli in two opposite directions. **(B,D)**, Cumulative curves of responses for the same stimuli, normalized to background firing rate. ^**^p < 0.01; ^***^p < 0.001.

Once the satellite leaves orbit it re-enters the Earth's atmosphere. At this point the organism experiences a hyper-gravity force until touchdown, about 45 min later. Readaptation likely begins during the re-entry phase and determines how quickly the organism recovers. The first and last nerve recordings of PF snails were made 14.75 and 26.13 h after landing, respectively. The PF snails were split into an early PF-e (<20 h after landing) and late PF-l (>20 h after landing) subgroups, and compared individually to the OL snails to reveal the process of readaptation of statocyst function. The statocyst response of PF-e subgroup was significantly larger than that of PF-l subgroup and most OL snails (Figure 9). Responses to tilt in the range (0.5–1.7) s were significantly larger in the two analyzed directions in PF-e subgroup (for c1 PF-e: 54.67 ± 4.16, PF-l: 36.75 ± 7.11, OL: 31 ± 2.13, DBH pairwise comparison against PF-e *p*_*adj*_ = 0.073 (n. s.—but *p*_*adj*_ = 0.03 with Tukey *post-hoc* test) for PF-l and *p*_*adj*_ = 0.002 for OL; for c3 (PF-e: 46.17 ± 3.64, PF-l: 29.75 ± 2.29, OL: 27.47 ± 1.89, DBH pairwise comparison against PF-e *p*_*adj*_ = 0.038 for PF-l and *p*_*adj*_ = 0.001 for OL).

The readaptation process in two different missions was compared by similarly splitting the electrophysiological data of Foton-M3 by time of preparation recording at 20 h after landing. Only two preparations were recorded before this critical time, but the overall picture is very similar to that obtained from Bion-M1 dataset (Figure S6). Statistics for such a small sample may be not reliable, but the required confidence level was reached for difference between early and lately recorded PF2007 subgroups, also as with control DK2007 group [range (1.3–2.2) s pairwise comparison against PF2007-e: 54 ± 2 vs. 32 ± 4.9 PF2007-l, *p*_*adj*_ = 0.04, DK2007: 21.88 ± 3.11, *p*_*adj*_ = 0.003; Tukey *post-hoc* test].

### Activity of vestibular efferent neurons

**Figure 10:**
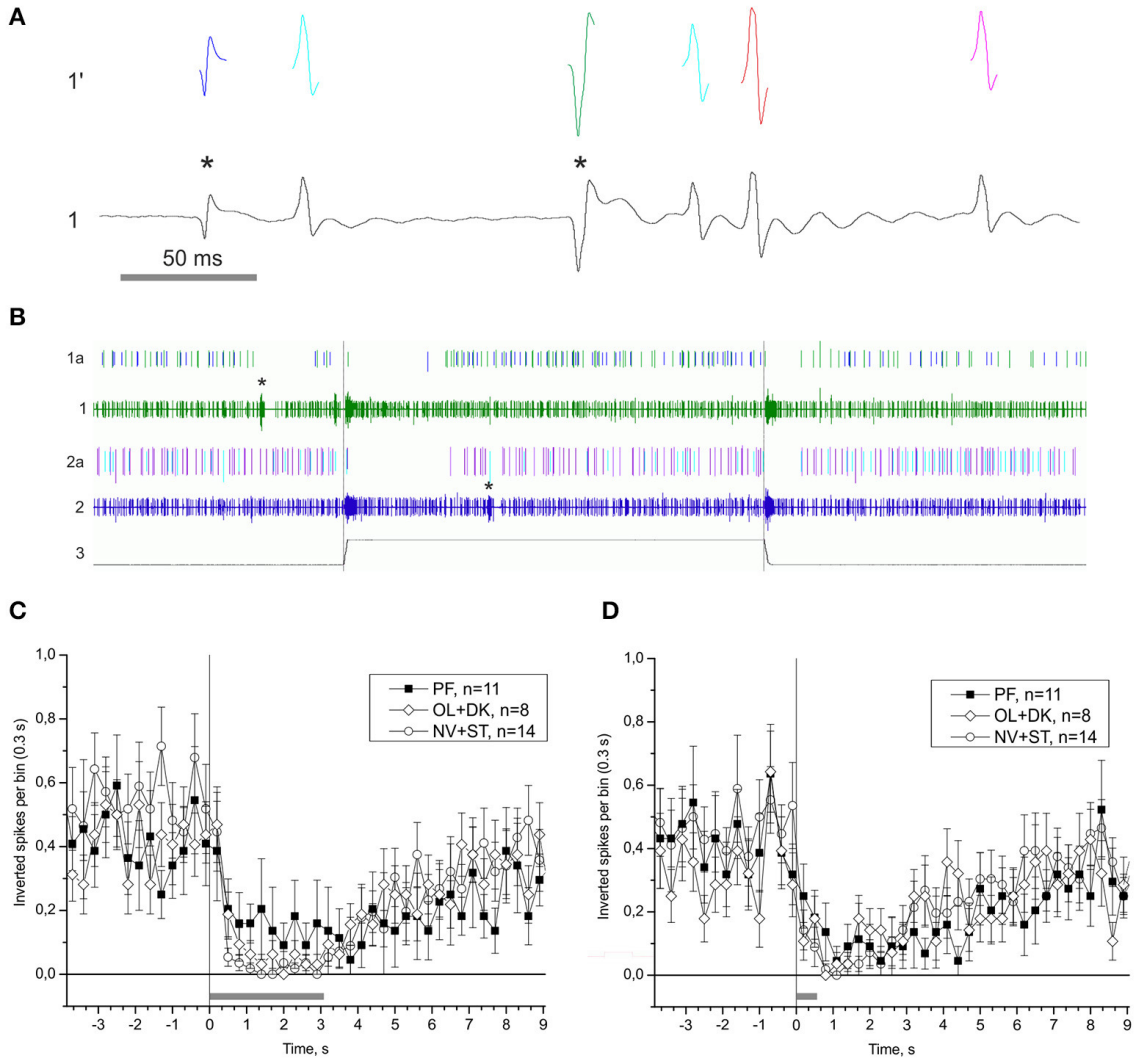
Single unit analysis of statocyst efferent firing rate. **(A)** Efferent action potentials to statocyst appear as inverted spikes in our extracellular records. 1—*n. vestibularis* record, 1′–spikes event derived from the record 1. Asterisks mark inverted spikes, which analyzed as separated channel. **(B)** Extracellular record of activity of the left and right statocysts: 1, *n. vestibularis sin*.; 2, *n. vestibularis dex*.; 1a and 2a, virtual channels derived by spike sorting of nerve activity, representing presumed efferent activity to the corresponding statocyst. In this example each nerve transmits spikes from two presumed efferent cells revealed by spike shape and shown in different colors. Note the OFF-responses in activity of efferents to spontaneous bursts in statocyst activity (marked by asterisks) and to bursts elicited by tilts (trace 3, 60 s between tilts). **(C,D)**, Averaged peri-stimulus curves of efferent firing rate to stimulus of 3,050 ms duration **(C)** and of 550 ms duration **(D)**. Tilt duration is given by the gray bar at the bottom of each plot.

One of the possible mechanisms of the statocyst activity modulation is by tuning the sensory cells via the central vestibular efferent fibers synapsing onto the statoreceptors. We were able to distinguish the activity of statocyst efferent neurons in our *n. vestibularis* records by spike sorting of single units with inverted polarity of spikes (i.e., traveling in centrifugal direction to statocyst). A possible efferent role in vestibular modulation was examined by comparing peri-stimulus curves of efferent activity in the different groups of snails (Figure 10). Efferent activity was found only in part of the preparations in any group, and in every case it was monotonous firing with OFF-responses to bursts of statocyst activity. No significant difference in efferent activity between groups was found [KW *p* = 0.78 (−4.9 − −1.9) s; KW *p* = 0.14 (0.2–2.9) s]. Such a pattern of activity and the lack of a significant correlation of it between the groups suggest a likely negligible influence of vestibular efferents on the background and induced activity of statocyst in our preparations. We studied also the effect of light ON- and OFF-stimuli in semi-intact preparations with intact eyes and found no light effect on activity of statocyst efferents and sensory cells (Figure S7).

### Effect of tilt direction on statocyst response and single unit analysis

Responses to tilt in the direction of head down to horizon position (c1) were larger than any other vestibular stimuli in preparations from all control groups and the PF group (see Figures S4, S8). The effect was more profound in the period immediately after the stimulus end (tonic response), and more obvious on cumulative plots (Figure S9). Experiments using a reversed order of stimuli (to control for conditioning) showed that there is some effect of the sequence (note the increased response to c4 direction on Figure S4); but even if the stimulus in c1 direction was presented last in the sequence, it generated the larger response of the statocyst.

**Figure 11:**
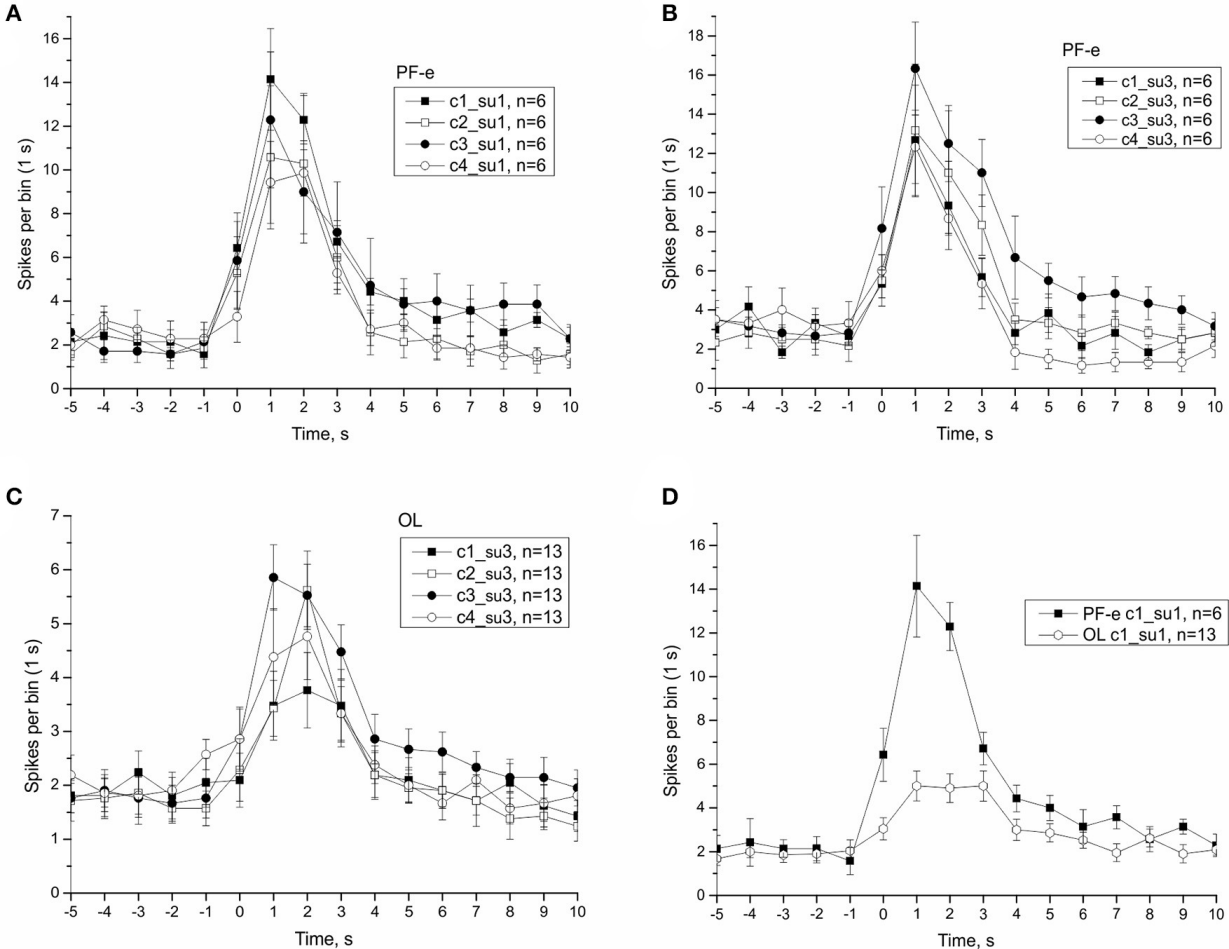
Single unit analysis of statocyst activity. **(A,D)**, Responses of single unit showed maximal response to stimulus c1. **(B,C)**, Responses of a separate single unit showed maximal response to stimulus c3.

**Figure 12:**
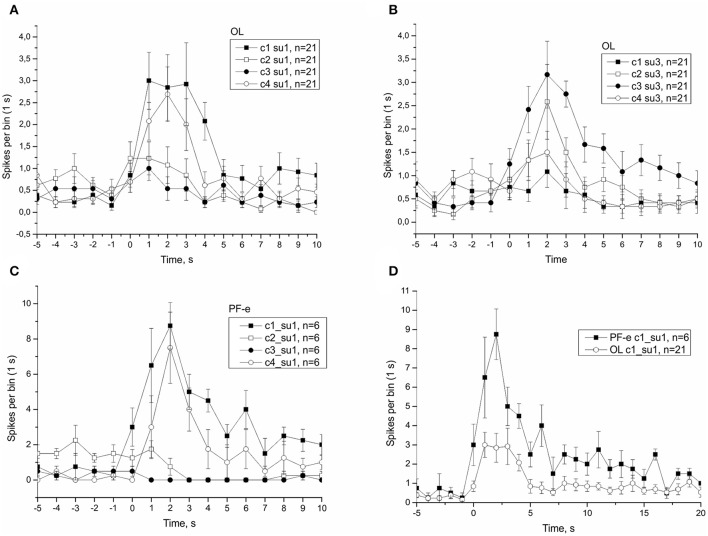
Single unit analysis of statocyst activity. **(A,C,D)**, Responses of single unit showed directional phasic response to stimulus c1. **(B)**, Responses of a separate single unit showed directional phasic response to stimulus c3.

**Figure 13:**
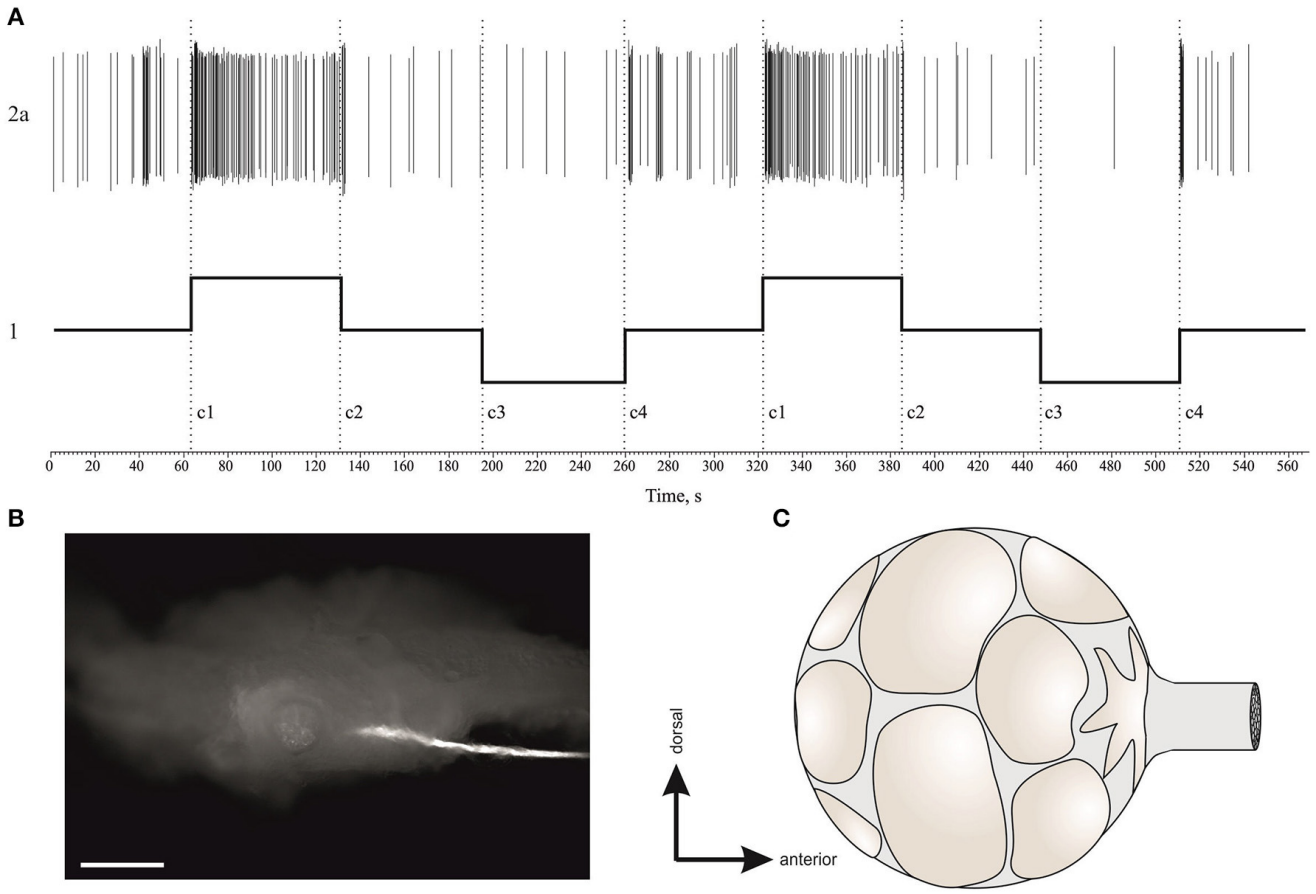
**(A)**, Example of a single unit in statocyst activity responding to head down (c1) tilt only. This single unit presumably represents the activity of the unpaired statoreceptor at the anterior pole of statocyst (star-shaped). **(B)**, The position of right statocyst on dorsal surface of right pedal ganglion, *n.vestibularis* are backfilled with carboxyfluorescein, calibration bar is 100 μm. **(C)**, Schematic drawing of statoreceptors' position in the statocyst, and the unpaired cell at anterior pole is star-shaped (based on data Gorgiladze et al., 2013).

In μG conditions statoreceptors are “unloaded” in contrast to normal gravity, and thus have less contact with the weight-lending statoconia. If an adaptation process occurs, the increase in sensitivity of statoreceptors may be expected. We analyzed the tilt responses of whole nerve activity at single unit level, namely the presumed single statoreceptors in two snail groups, PF-e and OL. The activity of single statoreceptors may signify an activation (ON-responses) or inhibition (OFF-responses) to vestibular stimuli, and varying phasic or tonic behavior might enrich the responses. All possible combinations of activity pattern were found in our recordings (Figure S10). First we plotted the averaged responses to different stimuli for two single units with a maximal phasic response (in 5 s) to either stimulus c1 or c3 (Figure 11). These single units contributed ~30% of the overall background firing level in the statocyst nerve, but showed a very weak sensitivity to vestibular stimuli (ON- responses to any stimulus), suggesting they presumably represented ventrally located statoreceptors, (Figures 11A–C). No increased tonic activity to c1 stimulus was found in these single units, so it cannot be the substrate of directional sensitivity we had found in net responses. Background firing rate of single units in PF-e group is similar to the one in OL group, but the responses are significantly larger (KW *p* < 0.001), Figure 11D. Another analyzed single unit's pattern was characterized by low or no background firing rate in the horizontal position and phasic-tonic responses to vestibular stimuli in only one direction (Figure 12). By the direction of its sensitivity the response likely originated from a frontally located statoreceptors for direction c1 Figures 12A,C,D) and from a caudally located cells for c3 (Figure 12B). These statoreceptors also showed dramatic difference between OL and PF-e groups, and no increased tonic activity for c1 direction. From single unit analysis only one explanation remains for increased responses for direction c1, observed in all groups. As *Helix* statocyst contains 13 statoreceptors, the unpaired cell, located at the anterior pole of the statocyst, drives the increase in response to head down tilt (c1). This cell responds to c1 stimulus and does not respond to any other stimulus presented (Figure 13).

## Discussion

### Behavioral studies

Upon entry into orbit the satellite is in a constant free fall back toward Earth and the traveler feels weightless. The neural gravity sensors that control normal balance and equilibrium are now forced to operate in a unique environment. The present experiments sought to identify the consequences of a 30-day orbital mission on the behavioral performance of snails challenged by a vestibular perturbation after return to normal 1G starting 14–15 h after landing. To account for possible environmental and habitat factors of unmanned spaceflights that can affect measured parameters of behavior other than those related to the state of weightlessness, we designed four control groups based on housing, food and water availability, and lighting, and included a landing-like simulation. In brief, we found that the behavioral phases most directly correlated with gravity sensation were the most affected by the vestibular perturbations tested as soon as the snails reached the laboratory after landing.

To distinguish μG-affected parameters, we made multiple group analysis of variance comparisons for each variable (*q.v*. Supplementary Materials). It reveals significant changes in the early phase of gravitaxis reaction parameters in the postflight (PF) group: the degree of tentacles withdrawal at the initial phase 0 was smaller (Figure 2B), and followed by shorter latency of tentacles protraction (T0, Figure 1C). Both findings were replicated in the data from an earlier 12-day Foton-M3 mission (T0 latency at the tendency level, phase 0 withdrawal highly significant; Figure S2). After the snail pivots to the head-up position (late T3-T4 phases), the length of tentacles becomes shorter in the PF group in comparison to 3 control groups (Figure 2). At the same time in the fourth control group, termed statocyst-affected “overloading” (landing simulation, OL), the tentacle length at T4 is significantly higher than that of the PF snails or any control snail, a finding that promotes a speculation on the opposite effect of overloading (hypergravity) and unloading (μG) exposure on the functions controlled by snail statocysts.

The cause of a lesser T2 latency in Foton-M3 postflight group in comparison to Bion-M1 PF group may be attributed to smaller snails used in Foton-M3 project (3–8 vs. 8–25 g, muscle mass/shell weight ratio is higher for young snails, allowing faster movements). We studied subgroups of young snails (3–8 g) in OL controls, but no correlation of T2 duration with weight was found (*q.v*. Supplementary Data). For the gravitaxis reaction, the snails affected by prolonged μG exposure show stronger correlations with snail weight than do control snails (Figure 3), thus suggesting a probable explanation of a shorter T2 phase in Foton-M3 postflight snails.

Snails belonging to the same genus of *Helix* (*H. aspersa*), the common garden snail, were exposed to increased gravitation by centrifugation for 16 and 32 days and showed an increased latency or slowing of T3 phase of gravitaxis (Popova and Boyle, 2015). In the data of Bion-M1 project opposite effects in PF snails to the control snails were not seen. There is no consistency between the different Bion-M1 control groups in this parameter, although two groups with most controlled conditions (DK and OL) did show shorter T1-T4 latencies than PF group. For Foton-M3 data (with comparable snail size used), all available latencies (up to T2) are shorter than in preflight controls, thus in agreement with the hypothesis that gravitaxis temporal parameters will change in an opposite fashion after μG than after hypergravity. No differences in T4 duration between μG PF and control groups of snails were found in the data of Bion-M1, also in agreement with the previous study of hypergravity influences and the assumption that the T4 phase may reflect more motor than vestibular function in snails (Popova and Boyle, 2015).

A correlation analysis of Bion-M1 behavioral data found T1-T4 phase latencies and durations were positively correlated among themselves in both PF and joined control snails, suggesting a stability of gravitaxis speed during the entire T1-T4 sequence (“slow snails are always slow”). The tentacle length in phases T1-T4 in both PF and control snails showed a positive inter-relation, suggesting the stability of withdrawal reaction during gravitaxis reaction (“afraid snails are always afraid”). Inter-tentacle angles are also positively correlated among themselves during the entire gravitaxis reaction (0-T4), although to a lesser extent than tentacle length and temporal parameters, probably due to the transient nature of this parameter. The tentacle lengths at T0 in the four merged control groups showed weak negative correlations with temporal parameters, together with T0 latency with tentacle length at many phases (Figure 3A). This finding may be interpreted that snails exhibiting more fear (retract tentacles shorter) complete the gravitaxis reaction more slowly. For PF snails such a correlation disappears (Figure 3B), supposedly because the disturbance of statocysts function shortly after landing results in less confidence to vestibular stimuli (habituation of response).

Vestibular stimuli sensed by statocysts were shown to be connected to tentacles withdrawal and generalized defensive reaction in snails (see Figure 6 here; Balaban, 1993). Pierantoni (1983) showed the connection of the vertical angle of tentacles and the function of snail statocysts using a spinning disk, and speculated that the vertical angle between the tentacles and the surface is associated with fear in the snail and will depend on the inclination of surface.

Inter-tentacle angle in land snails has been investigated mostly in the context of olfactory orientation (Chase and Croll, 1981; Davis, 2004; Davis-Berg, 2012), although tentacle connection with collumellar musculature and hydroskeleton may provide some mechanistic relation of the inter-tentacle angle with muscular or hydraulic tonus of snail; for example, in experiments with predatory snails inter-tentacle angle seems to be correlated with speed of snail crawling (Davis, 2004; Davis-Berg, 2012). In our experiments inter-tentacle angles were measured from the screenshots of different phases of gravitaxis reaction, and the analysis revealed a tendency of the angle to become narrower in late phases in all groups of Bion-M1 and Foton-M3 experiments (Figures S1A, S2C). No specific effects of prolonged μG (PF snails) or short centrifugation (OL snails) on inter-tentacle angle were found in Bion-M1 experiments, although a decrease of inter-tentacle angle in all phases of gravitaxis was found in Foton-M3 experiment (Figure S2C). A possible cause for this discrepancy is a difference of experimental design: repeated testing of snails in Foton-M3 project as opposed to only one test in Bion-M1 snails.

Both PF and OL groups show differences in opposite directions in comparison to three other controls in tentacle length at late gravitaxis phase T4 (Figure 2A). Both groups are statocyst-affected, but in an opposite direction, as OL snails were shortly centrifuged, and PF snails were exposed to prolonged μG (plus very similar overloads as OL during landing). In our physiological experiments no changes in background statocyst activity were found, but the response to vestibular stimulation was significantly stronger in PF group in comparison to control groups, and responses of OL group were weaker compared to other control groups (Figure 7). Such physiological differences may underlie the observed behavioral differences.

### Transcriptome analysis of statocysts

The changes in neural cells' morphology, biochemistry and genes expression under space flight conditions are widely accepted (Honda et al., 2012; Pani et al., 2013; Ranjan et al., 2014; Tsybko et al., 2015; Wang et al., 2016; Porseva et al., 2017). Among the most significant differentially expressed genes in PF snails are the neuronal specific precursors of neuropeptides, neurotransmitter receptor subunits, and other genes involved in cell signaling. Gene expression of both the pedal and FMRF-amide peptides were tested by *in situ* hybridization in earlier Foton studies (Balaban et al., 2011), and were found to be differentially expressed in Bion-M1 PF snails in comparison to ST control snails. In the present Bion-M1 study the genes for these peptides were significantly downregulated in PF-e samples (see Figure 5 and Table S1). In Foton studies preproFMRF-amide gene was found unaltered in control and postflight statocysts using the ISH technique, and preproHPep was found to be upregulated in certain receptor cells. Such discrepancy with Bion-M1 may be explained at least in part by different sensitivity of methods used and also a different *Helix* species (*Helix aspersa* L.) was used in Foton-M3 ISH experiment.

### Adaptation to spaceflight and readaptation process

The present study confirmed the electrophysiological findings observed in two earlier Foton satellite missions (Balaban et al., 2011). This study also benefitted from the earlier Foton missions: an earlier animal return to the lab after touchdown was achieved (chartered jet and police escort from the airport) and the experimental protocols evolved to capture as much data as possible in the shortest period of time. Statocyst responses in PF snails of Bion-M1 were comparable to their counterparts in Foton-M3 (see Figure 8), despite more than a 2-fold difference in mission duration. The finding of a postflight hypersensitivity to tilt was confirmed at the level of the whole nerve and single statoreceptor analyses (see Figures 11–13). The statocyst background rate remained unchanged as in the 2007 mission. These findings suggest we measured on a plateau portion of the sensitivity adaptation process, and that the mechanisms underlying in-flight changes, i.e., the μG-induced influence, in statocyst responses are established before 12 days in orbit. We were able to identify the critical time of readaptation to Earth 1G, at ~20 h after landing. Interestingly, hypersensitivity of sensory neurons after spaceflight was found not only in vestibular system, but also in mechanosensory receptors of human skin (Lowrey et al., 2014).

The response sensitivity of utricular afferents in the vertebrate toadfish, *Opsanus tau*, taken post-landing of the orbiters STS-90 (NeuroLab; 16 days in orbit) and STS-95 (9 days in orbit) missions revealed an induced profound hypersensitivity to translational acceleration tests (Boyle et al., 2001). Recordings were also made in separate populations of afferents at numerous time periods after landing to track the rate of recovery. Although some afferents remained hypersensitive, the population as a whole recovered within 24–36 h comparable to readaptation time of snail sensory cells and experienced astronauts, and a bit shorter than a 2–3 day space motion sickness period reported in astronauts during 1G readaptation following 6-month manned missions (Heer and Paloski, 2006).

It can be argued in the vertebrate that the rate of adaptation to spaceflight and its recovery after return to Earth are more consistent with a restructuring of the synaptic organization between the hair cells and the afferents, such as adjusting the number of synaptic ribbons or bodies (Ross, 2000), than a restructuring the weight-lending otolith (see below). The toadfish study could not identify whether the hypersensitivity originated in the hair cell, the hair cell-afferent complex, or postsynaptically in the afferent itself. In our Foton and Bion-M1 studies in the snail statocyst we recorded directly from the output of the statoreceptors themselves. Although it is tempting to speculate that the hair cells in vertebrates are also the targets of the sensitivity adjustment to altered gravity loads, direct evidence is still lacking.

Vestibular sensitivity in the statocyst of the garden snail was recently studied following exposure to hyper-gravity conditions using centrifugation (Popova and Boyle, 2015). A significant decrease in response sensitivity to head-down tilt was detected after 16- and 32-day (but not 4-day) exposures to a 1.4G [resultant = √1G (Earth)^2^ + 1G (centripetal)^2^]. After a 7-day recovery period in normal 1G the responses returned to normal. In accordance with the present study the prevalent gravity level appears represented as a continuum in statocyst function over the measured periods of time. Critical data that are missing and subject to future investigations are the initial responses of the statocyst and organism to an abrupt change in gravity load, the functional and structural consequences of long-term exposure to altered gravity, and the time course of recovery of function as a function of load and duration of exposure.

### Light cycle influences on the statocyst response

Activity recorded in the lip sensory nerve in semi-intact *Helix* preparations can reflect the circadian phase to which the snail is adapted (Voss et al., 1997). In that study chemical stimuli presented in the light elicited an increased neural response when they corresponded in time to the snail's normal active phase of behavior, *viz*. scotophase. This species is known to have an endogenous circadian oscillator entrained by light (Bailey and Lazaridou-Dimitriadou, 1986; Attia, 2004), and the circadian pattern of locomotor activity remains with a period of ~25 h in constant darkness for at least 20 days (Bailey and Lazaridou-Dimitriadou, 1986).

The interpretation of the present results must take the influences of the light-dark cycle into consideration. Bion-M1 PF snails arrived at the lab in the evening, and the physiological recordings were mostly completed that night. The ST and NV control snails on the other hand were recorded during the daytime. If the statocyst contains a self-sustained peripheral circadian oscillator, it may affect the physiological responses we measured. To exclude such a possibility we performed an additional experiment that confirmed the statocyst response was independent of the circadian time in our preparation (Figure S5). However, the two Bion-M1 control groups with controlled dark conditions (DK and OL, 30 days in dark, recorded in scotophase) and the Foton-M3 control group (DK2007, 12 days in dark, recorded in daytime) show a decreased statocyst response in comparison to ST (natural light cycle, ~16L:8D) and NV (12:12 h LD cycle) control groups (see Figures 7, 8). Despite housed in the dark during the missions, Bion-M1 PF and PF2007 snails showed an increased statoreceptor sensitivity to tilt stimuli, whether recorded at night or during the daytime. This increase sensitivity found only in PF snails was presumably the result of the space environment and not from influences of the housing or time of recording. How these external factors interact to shape the statocyst response is unclear, as well as whether or not the interaction occurs at the same site of plasticity. Another route through which the light-dark cycle might modulate the statocyst activity is via the vestibular efferent fibers (Williamson, 1986; Janse et al., 1988; Tsubata et al., 2003; Sakakibara et al., 2005), that may be entrained by a central circadian oscillator (Attia, 2004). We compared the peri-stimulus curves of efferent activity and found no significant difference between PF and control snails (see Figure 10), thereby excluding the efferent neurons of dynamically modulating the statocyst response on a tilt-by-tilt basis. Whether the substrate for an effect of light-dark cycle is within the statocyst itself or elsewhere remains to be determined.

### Inertial mass as adaptation mechanism

A widely discussed mechanism of adaptation to the μG environment is an adjustment of the weight of statoconia (invertebrates) or otoconia (vertebrates) (Wiederhold et al., 2000; Edelmann, 2002; Gorgiladze, 2002). Deposition of calcium carbonate will affect a change in the mass, but not the weight, in space, but would reveal itself after return to the normal gravity. Ultrastructural studies in *Helix* found statoconia growth, i.e., size and amount, after long-term exposures to μG on Mir and ISS space stations (Gorgiladze, 2002; Gorgiladze et al., 2011). In Gorgiladze's (2002) study, statoconia size increased with μG exposures between 40 and 148 days, but for shorter exposures the data less clear as the statoconia size after 14 days was larger than after 31 days. Statoconia growth was reversed after return to 1G in about 30 days. In their postflight snails the statoconia were often incrustated by “flakes” or covered by a layer of distinctive crust indicating a deposition of calcium carbonate. Our limited morphological data from Bion-M1 postflight statoconia did not show any of these characteristic features (see Figure 4). However, caution is needed because our sample size was restricted to a single animal in 3 groups and the flight durations were not similar. This compensatory process requires more examination. One more factor is pertinent to the different results reported in *Helix* studies: in our study adult animals (20–25 g) were used, whereas in the previous studies snails were either juvenile (0.3–2 g) or young adults (9–15 g). The effect of statoconia growth in μG was more pronounced in young snails (Gorgiladze, 2002), and was insignificant in adult fish otoconia (Wiederhold et al., 2003).

In addition, our physiological data showed no difference in the magnitude of the sensitivity changes between the postflight groups of our separate missions having different duration of μG exposure (shown in Figure 8). Thus, there is no substantive evidence for a mechanism inducing a change in statoconia mass for snails exposed to μG for less than a month. For the process of readaptation to normal gravity, postflight groups of Bion-M1 and Foton-M3 studies both divided into two distinct subgroups by the critical time of recording 20 h after landing. It is even less likely that changes in statoconia play any role in this readaptation process of such short duration. Taking into account 20 h as critical time of readaptation, neural plasticity of statocyst sensory statoreceptors themselves, central circuits, or both might be better candidates.

### Role of efferent vestibular neurons

The vertebrate inner ear sensory organs receive centripetal innervation of their hair cells (and the dendrites of afferent neurons) by centrally located efferent neurons. This strategy is also present in snail statocyst: the statocyst nerve contains the axons of the statoreceptors as well as axons of neurons from the cerebral ganglion on each side. How the efferent neurons influence the gravity and acceleration sensations during and after space flight is unknown. Assuming the efferent innervation serves to tune the receptor's response to acceleration in a behaviorally relevant context (Goldberg et al., 2002; Boyle et al., 2009; Rabbitt et al., 2010), μG might trigger a shut down of the efferent system to allow an amplification of any input, now weak or absent, signal. If so, this might temporarily lead to a hypersensitivity in snail statoreceptors. In an attempt to address this key issue, we analyzed the firing behavior of presumed efferent neurons to tilt and found it was monotonous within each examined group of snails. Thus, direct evidence that the efferents play a role in the postflight statoreceptor hypersensitivity was not found. We cannot rule out however that the efferent neurons were studied in a behaviorally irrelevant context, namely in the isolated neural preparation and not in the intact animal, and postflight but not inflight.

### Directionality of response and single unit analysis

Previously we reported (Balaban et al., 2011) that the differential sensitivity of statocyst responses to stimuli of different directions (c1 > c2, c3, c4) seen in control snails was lost in the Foton-M3 postflight snails, and we hypothesized a weakening of directional selectivity might occur immediately after spaceflight. Here, we replicated this finding in the Bion-M1 control groups, but the PF snails of Bion-M1 remained spatial tuned to preferred directions (Figures S8, S9). Because of the robustness of the new results we reject our earlier hypothesis that spaceflight influences directional selectivity. Directional selectivity was also maintained in vertebrate otolith afferents with hypersensitivity after spaceflight (Boyle et al., 2001) and in centrifuged snails having reduced overall tilt sensitivity (Popova and Boyle, 2015). We consider this spatial tuning as a fundamental property of statocyst function and not readily susceptible to plastic changes. By spike sorting of the statocyst nerve activity we were able to distinguish the activity of single sensory cells. In some cases the cell activity could identify its possible location in the statocyst, like ventrally located ciliary cells or frontally from caudally located cells. We tried in all cases to find the directionality at the single unit level, but the most active cells in our records frequently showed little to no direction tuning to tilt presented in our protocol; these cells are presumably ventrally located in the statocyst and code more the position of animal in space than stimulus direction (see Figure 11). In the nudibranch *Hermissenda* the 13 statocyst sensory cells have cilia organized for multidirectional sensitivity (Kuzirian et al., 1981). In *Helix* some statocyst sensory cells might share a form of this organization in functional terms. Using single unit analysis we identified a few sensory cells that responded to only one of the two tested directions of tilt with a phasic-tonic reaction (see Figure 12). If we consider this to represent a multidirectional sensitivity of a statoreceptor, this type of response might be constructed in frontally and caudally located statoreceptors by a physical contact only with the edge of the inertial mass in the horizontal position of statocyst. Activity of these cells allows the CNS to decode the direction of stimuli from the statocyst output. Comparing the activity of identified statoreceptors, position-coding ventrally located cells form between 50 and 80% of the statocyst output (for OL: ~20 spikes/s, for PF-e: ~30 spikes/s during tilt), and direction-coding cells contribute ~15% of the net response.

The only viable candidate to explain the existence of directional response with higher sensitivity (“head down,” c1) is the unpaired sensory neuron at the anterior pole of the statocyst (see Figure 13). Responding to perturbations of vertical orientation of the organism, including human, in the “head down” direction are of critical importance for self-protection, and the unpaired sensory neuron at the anterior pole of the statocyst adds to the net information signal of tilt stimuli in this biologically meaningful direction.

Marine mollusks require a fast and reliable recognition of spatial orientation from variable external cues to maintain proper balance and 3D navigation in the sea, and thus their statocyst is the most studied among invertebrates (Wood and Von Baumgarten, 1972; Alkon, 1975; Janse, 1983; Budelmann and Williamson, 1994; Deliagina et al., 1998). In freshwater species, pulmonate *Lymnaea*, the statocyst output is important for periodical trips to the surface for breathing (Janse, 1982). Janse et al. (1988) compared the statoreceptors firing rate and gain in marine *Aplysia* and freshwater *Lymnaea* and speculated that the difference in ecology of the animals leads to a more sensitive statocyst in *Aplysia*. For land snails vestibular function is important for gravitaxis reaction, when the animals climb to elevated positions for diurnal rest in hot and dry day-time, and when returning back to the soil at dusk (Cowie, 1985; Aubry et al., 2006; Di Lellis et al., 2012). Another obvious biological significance of statocyst function is its role in the withdrawal reaction, as a sudden change of the body position may preclude an attack of a predator. In agreement with ecology of land snails, our physiological data show that the *Helix* statocyst operates more for coding the orientation of animal's body than fast changes in its direction.

## Author contributions

Research concept and design: NA, AM, VI, IZ, RB, and PB. Collection and/or assembly of data: NA, AV, MR, TK, AM, AZ, VI, ML, IZ, YP, RB, and PB. Behavior analysis and interpretation: NA, AV, MR, and TK. Electrophysiology: NA, MR, AM, AZ, ML, IZ. SEM: NA, IN, and RB. RNA-Seq and bioinformatics: SV, PK, EC, AK, and LU. Statistical analysis: NA. Writing the article: NA and RB. Critical revision of the article: NA, IZ, RB, and PB.

### Conflict of interest statement

The authors declare that the research was conducted in the absence of any commercial or financial relationships that could be construed as a potential conflict of interest.

## Supplementary material

The Supplementary Material for this article can be found online at: https://www.frontiersin.org/articles/10.3389/fncel.2017.00348/full#supplementary-material and at OSF https://osf.io/v5brf/

## Abbreviations

PF: BION M-1 postflight group of snails
NV: Naïve group of snails
ST: Starvation control group of snails
DK: Dark control group of snails
OL: Overload control group of snails
KW: Kruskal-wallis test of the null of equality of all 5 groups
DBH, Dunn's method of *post-hoc* pairwise tests: *p*-values after Benjamini-Hochberg adjustment for multiple comparisons against the PF group
CI: Confidence interval
n.s.: Non-significant
SEM: Scanning electron microscopy
PCA: Principal component analysis
FPKM: Fragments per kilobase million.
