# Supplementary material for: Adaptive Changes in the Vestibular System of Land Snail to a 30-Day Spaceflight and Readaptation on Return to Earth
Source: Front Cell Neurosci. 2017 Nov 1;11:348. doi: 10.3389/fncel.2017.00348 (PMC5672023; doi:10.3389/fncel.2017.00348)
Supplement: Supplementary file 1 [file Image1.PDF]

Supplementary figures

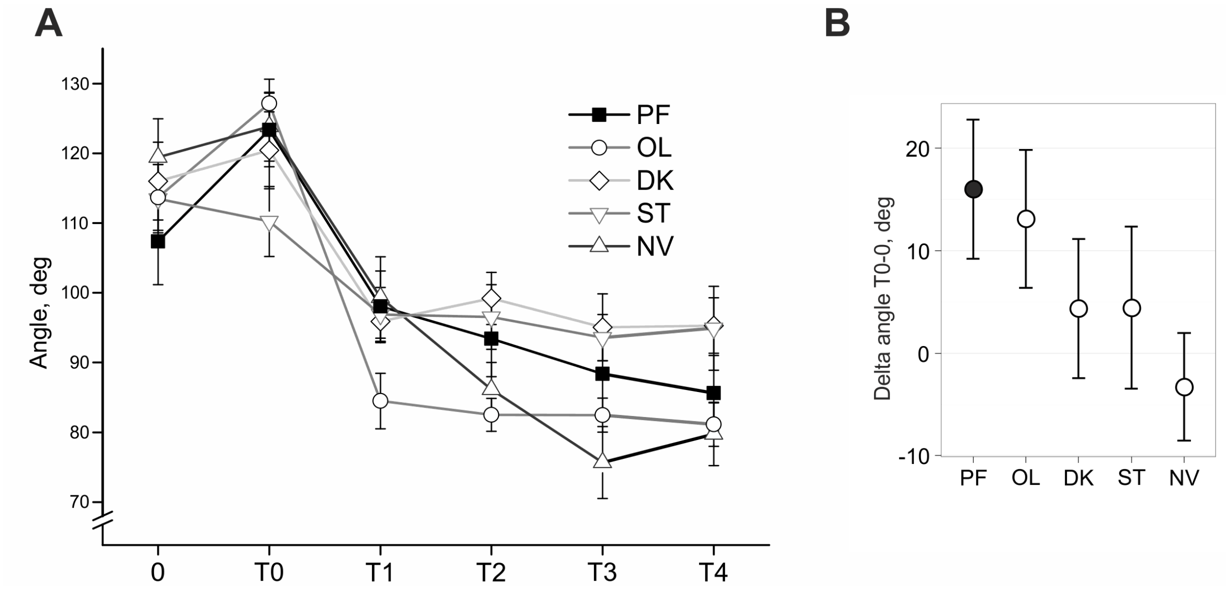

Fig. S1. Inter-tentacles angle shows no difference between groups (A). B — Angle change from phase 0 to T0 also shows significant difference only between NV-OL and NV-PF groups (mean and 95% CI).

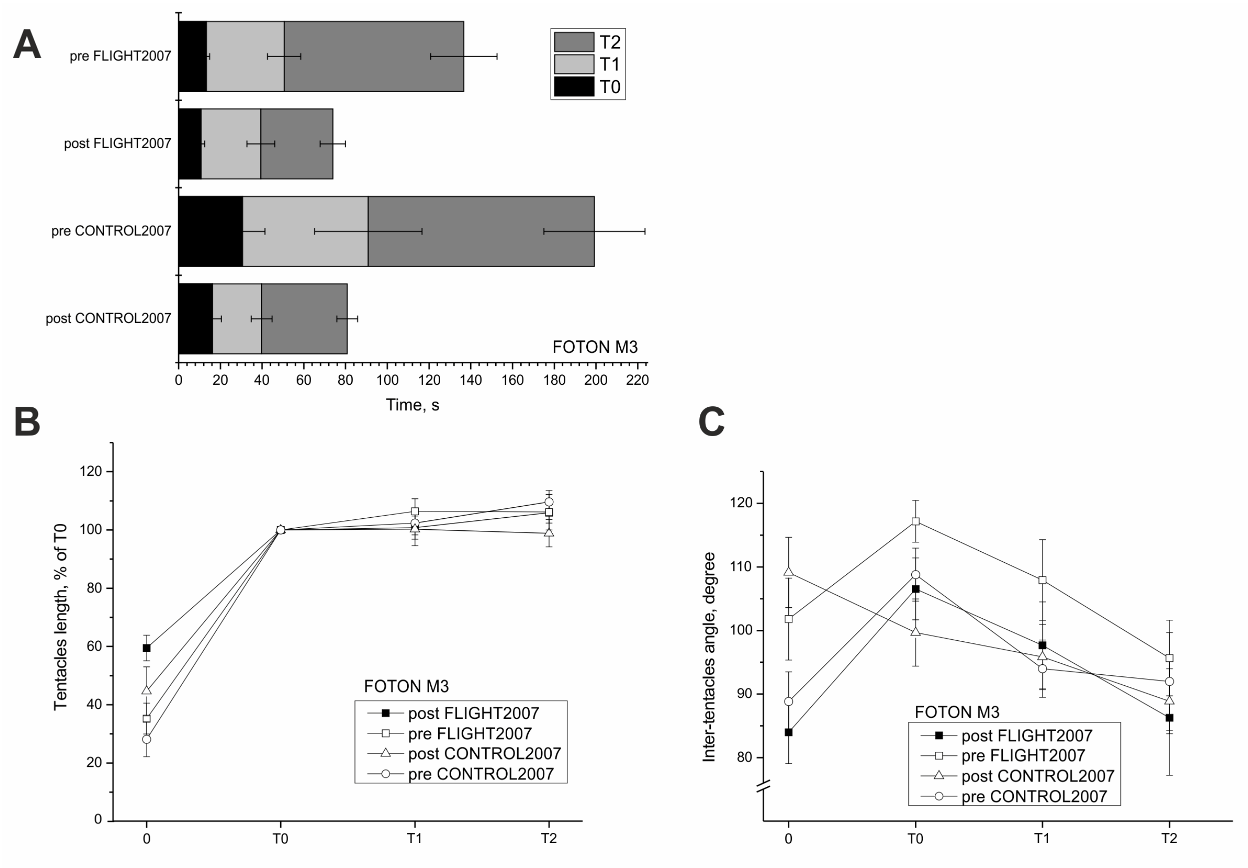

Fig. S2. Reanalysis of Foton M3 2007 behavioral data. Temporal dynamics (A), tentacles withdrawal (B), and inter-tentacles angles (C).

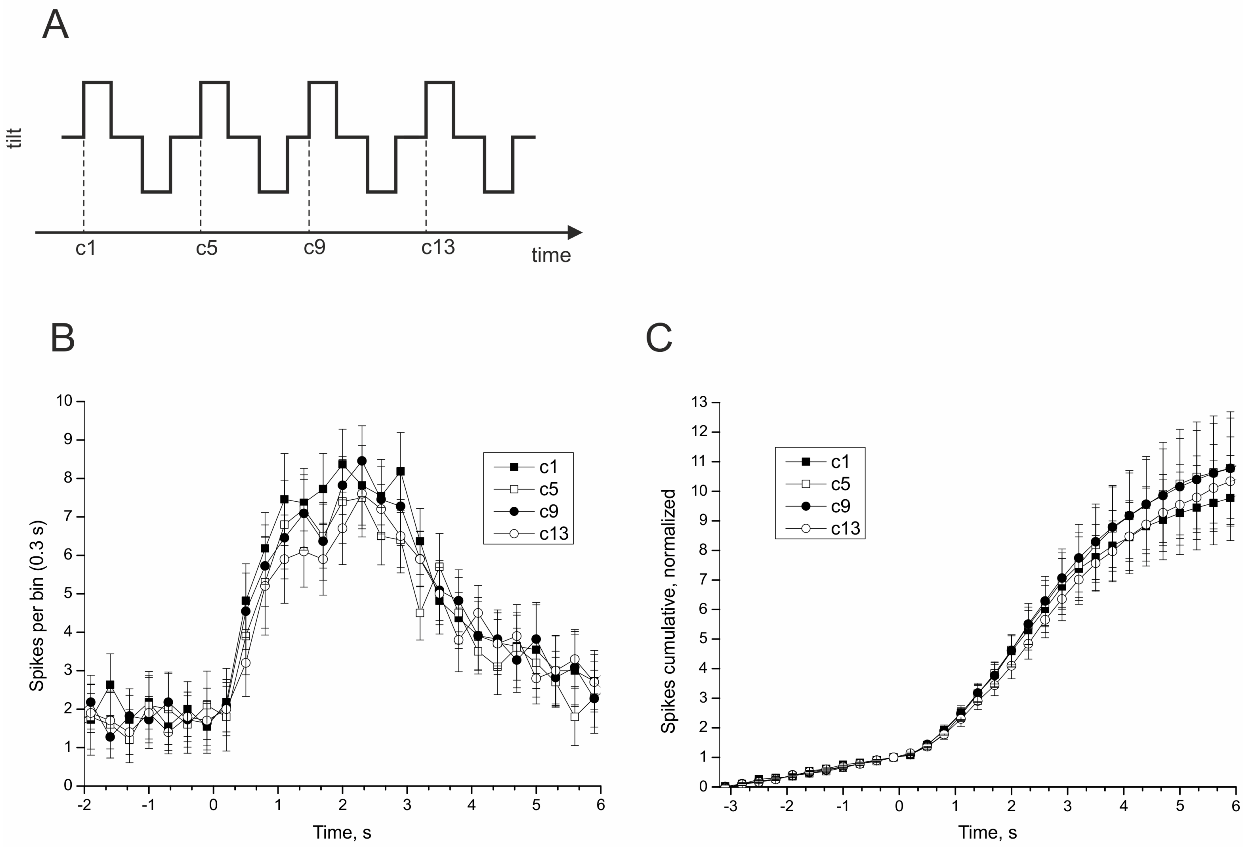

Fig. S3. Statocyst responses to long sequence of stimuli with 1 min inter-stimulus interval. A — order of stimuli, B, C — statocyst responses varied insignificantly in the middle of stimulus panel. For the main protocol first four stimuli in sequence were thrown out and second cycle of repeating stimuli was used for analysis.

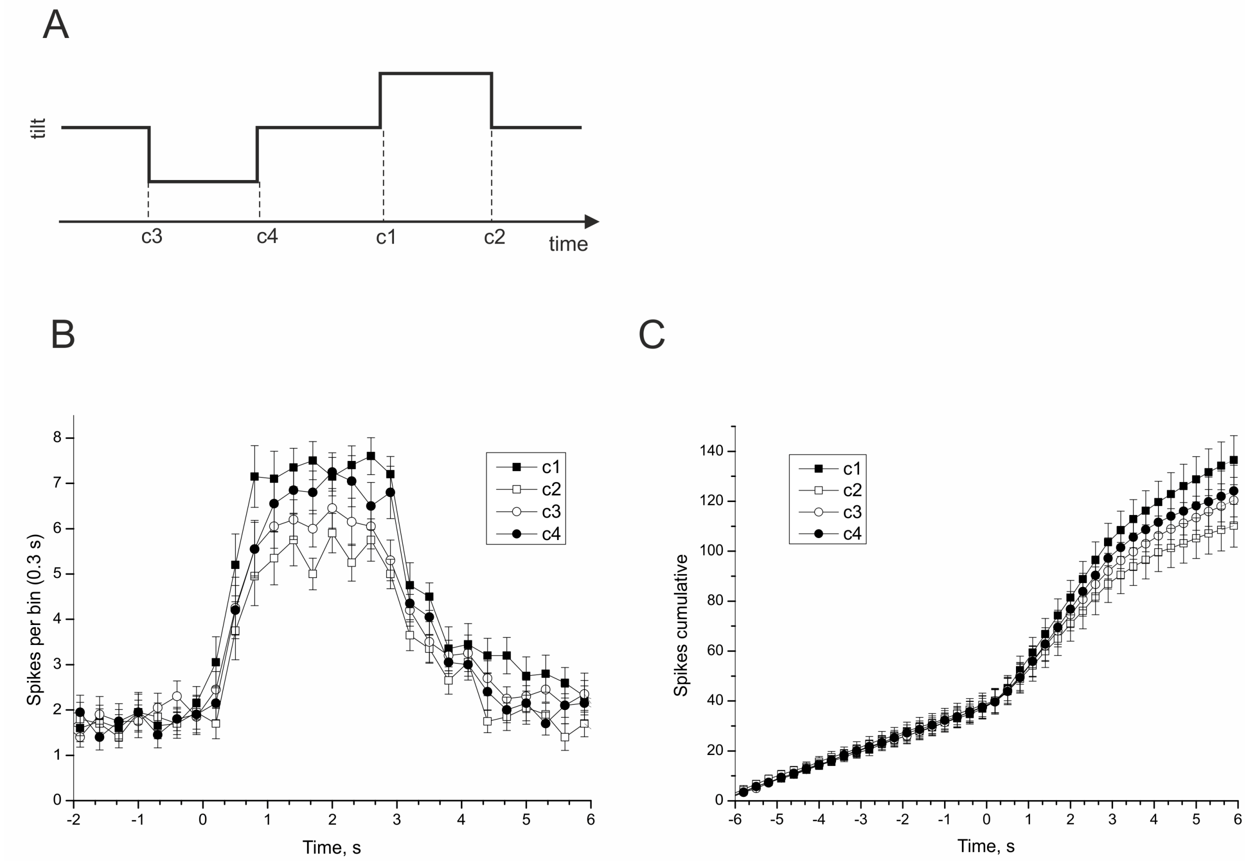

Fig. S4. Statocyst responses to reversed order of stimuli. A — order of stimuli, labels did not changed. B, C — statocyst responses; larger response is to c1 stimulus despite of changed order of stimuli.

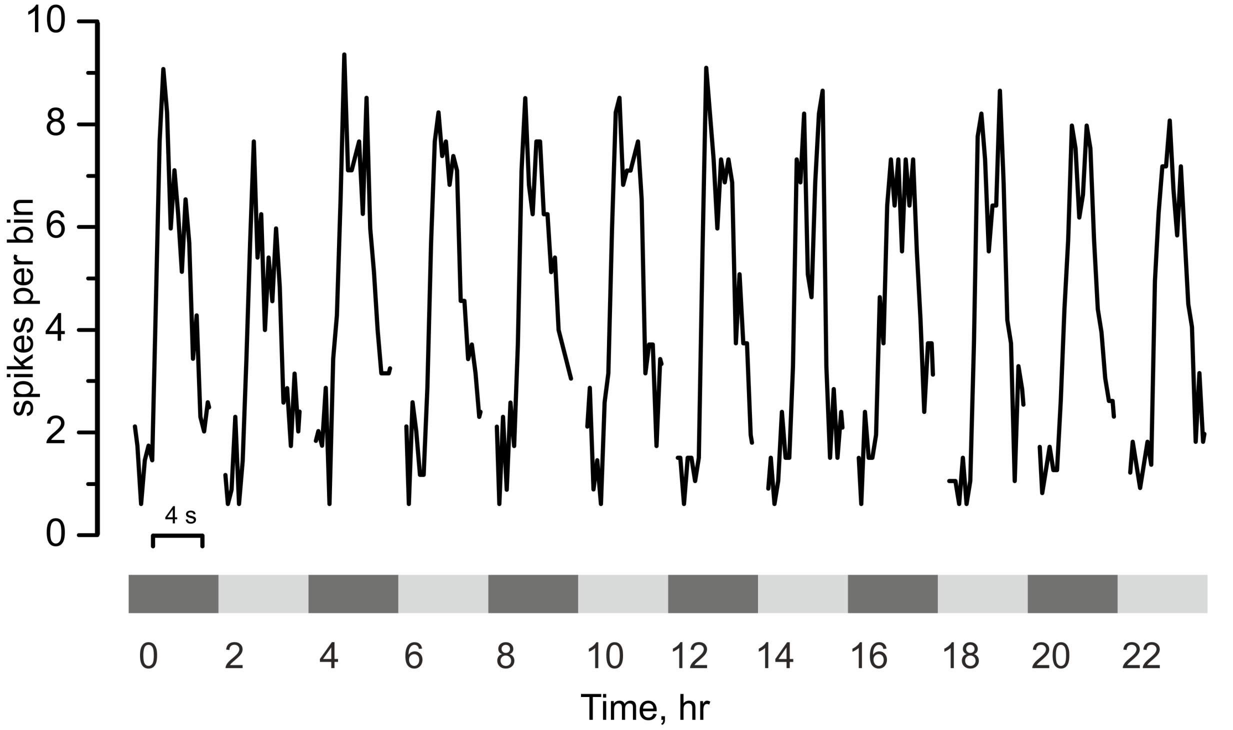

Fig. S5. No circadian variability of *n. vestibularis* responses to tilt was found in used CNS preparations. Automated system was made to present 13 min cycle of 12 stimuli every half hour during day and night. Eleven preparations were used to cover each period at least thrice.

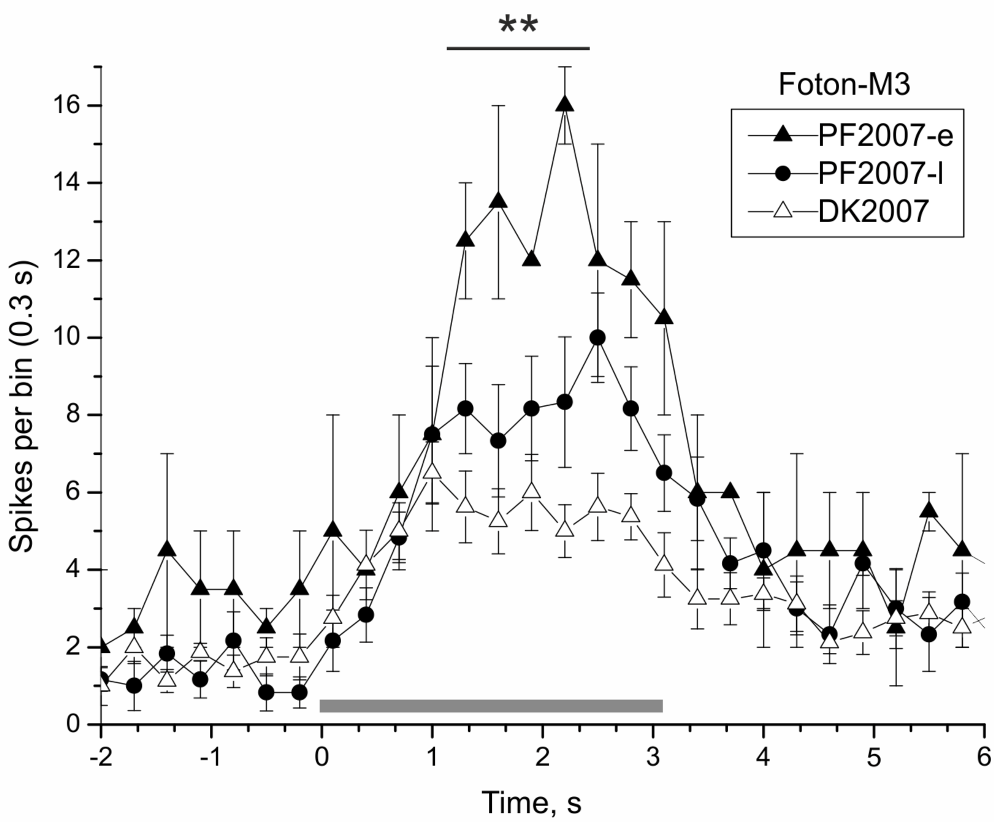

Fig. S6. Splitting postflight group of Foton-M3 by time of recordings reveal re-adaptation process. Averaged statocysts responses to vestibular stimuli; early recorded PF2007-e subgroup shows larger magnitude of responses.

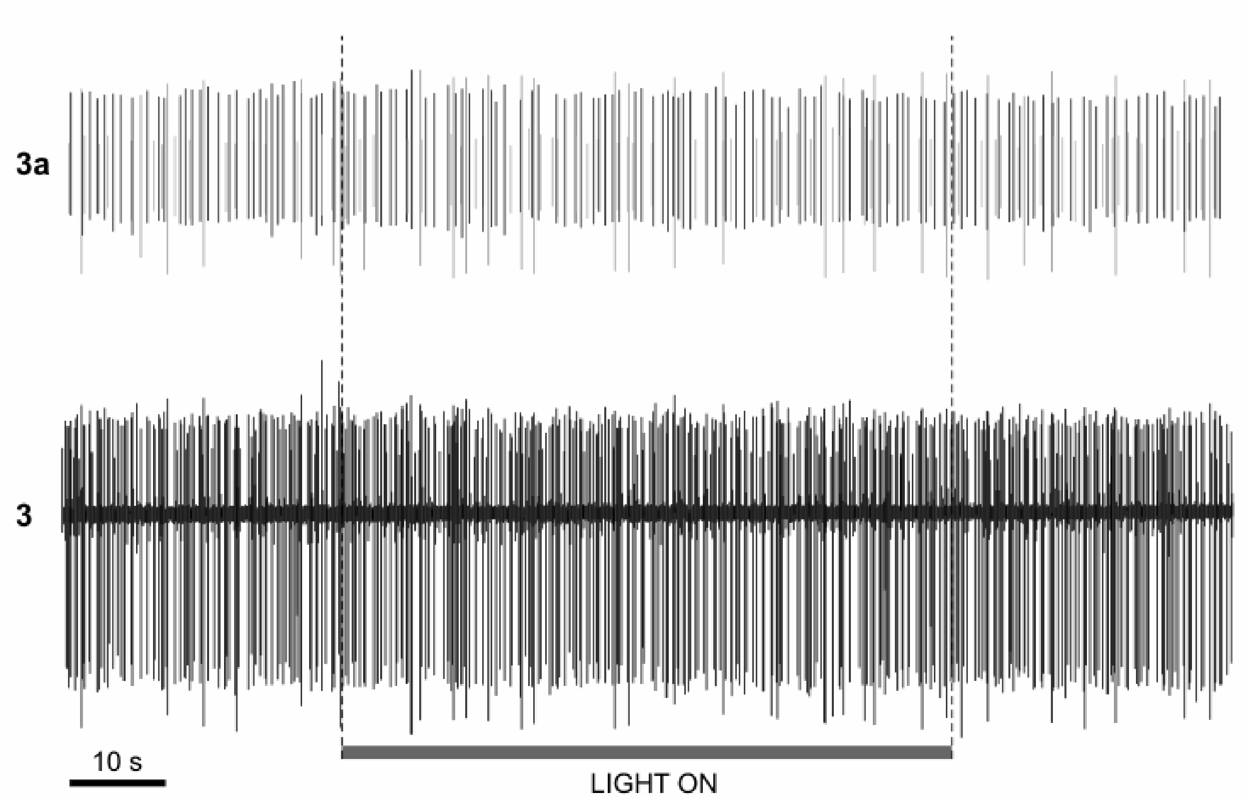

Fig. S7. Light does not affect the activity of statocyst sensory neurons and efferents. Trace 3 represent the overall nerve activity (high-amplitude spikes comes from statocyst); trace 3a derived from trace 3 is represent efferent spikes going to statocyst. Gray bar behind is represent time of eyes illumination by light (warm white, 70 W bulb via light guide).

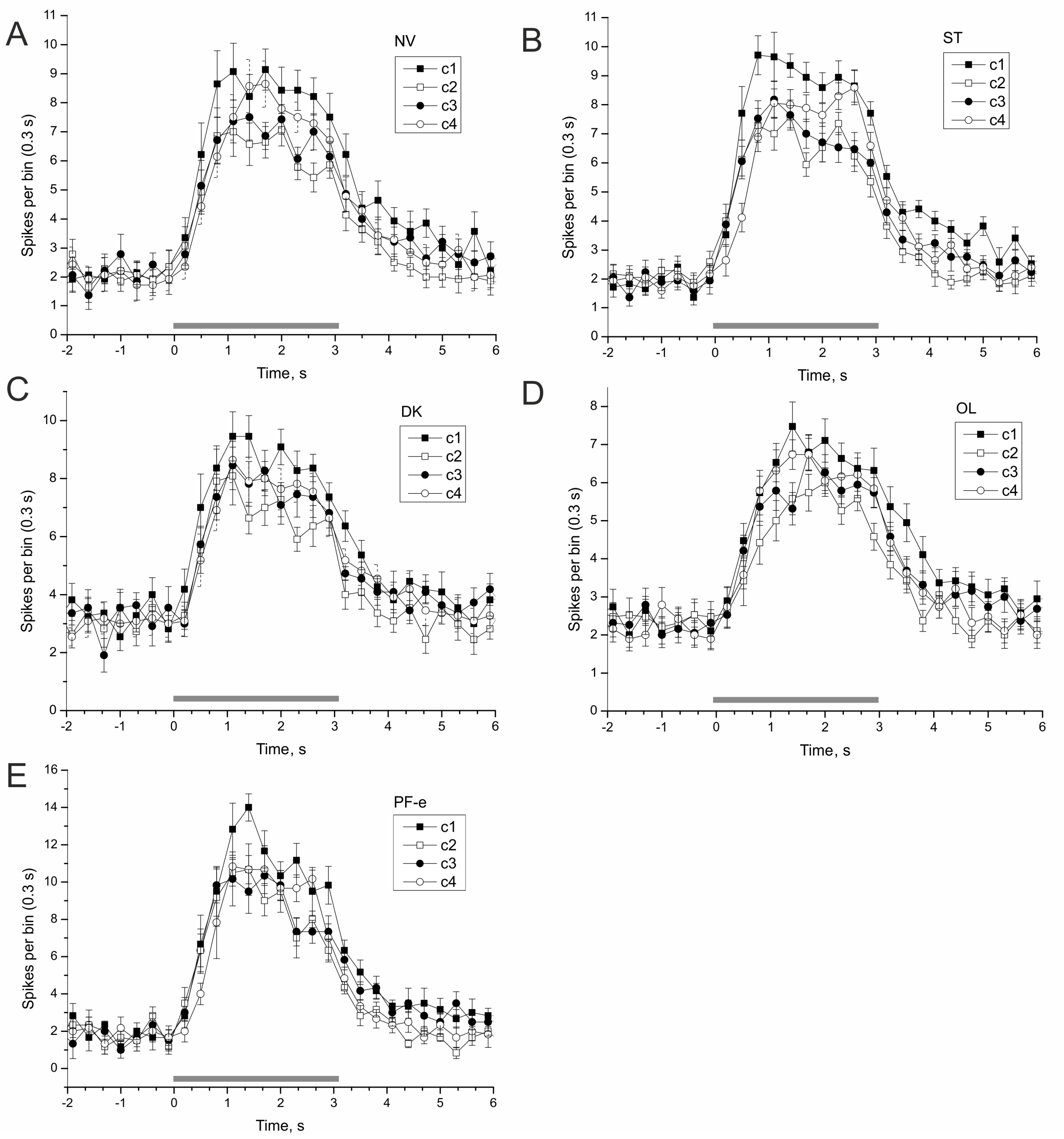

Fig. S8. Peri-stimulus curves of statocyst responses to vestibular stimuli in different direction for all groups of snails. Order of presentation and designation of stimuli as in Fig. 5A. Stimulus shown as gray bar at the bottom of the plots.

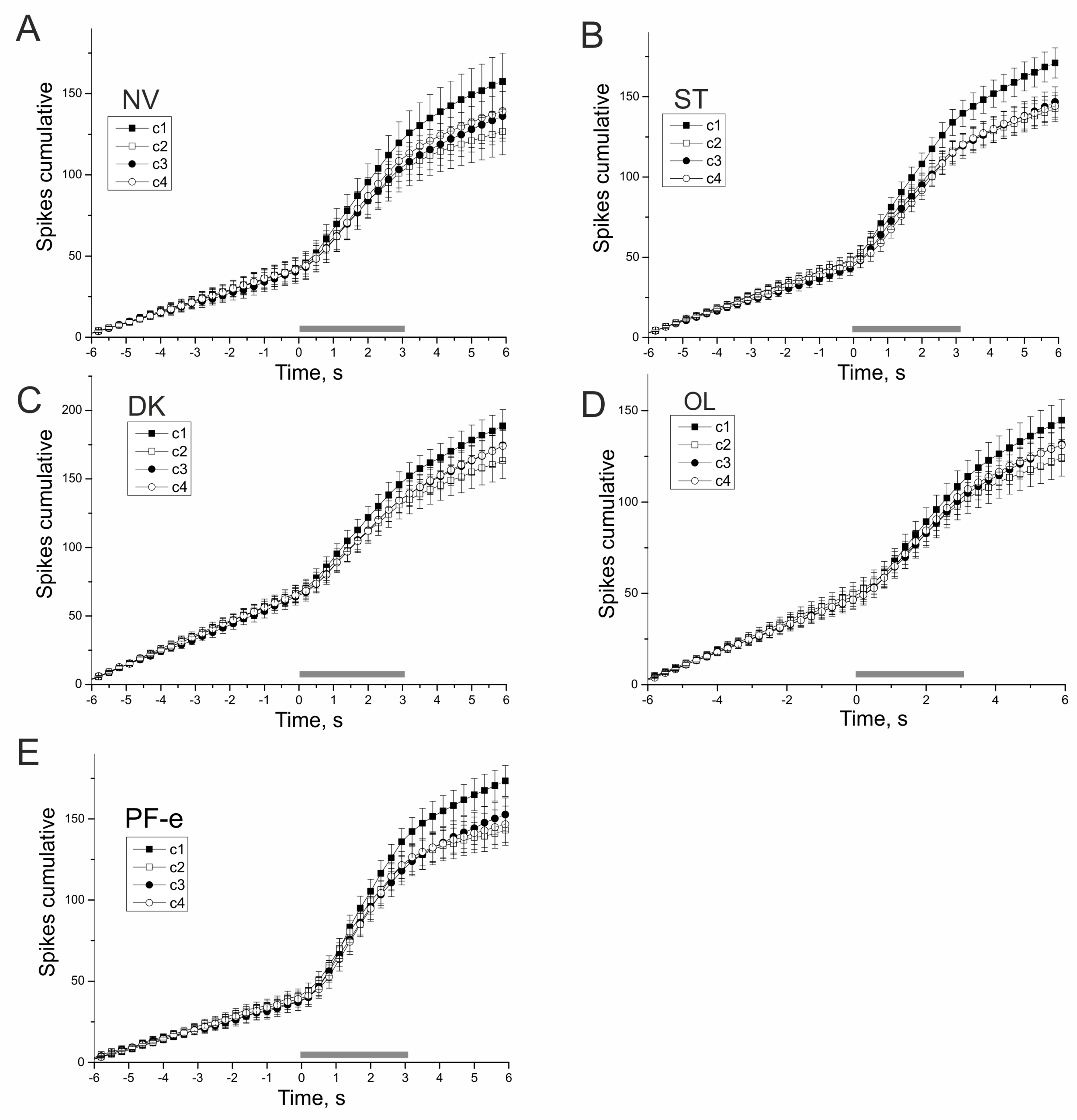

Fig. S9. Cumulative peri-stimulus curves of statocyst responses to vestibular stimuli in different directions for all groups of snails. Order of presentation and designation of stimuli as in Fig. 5A. Stimulus shown as gray bar at the bottom of the plots.

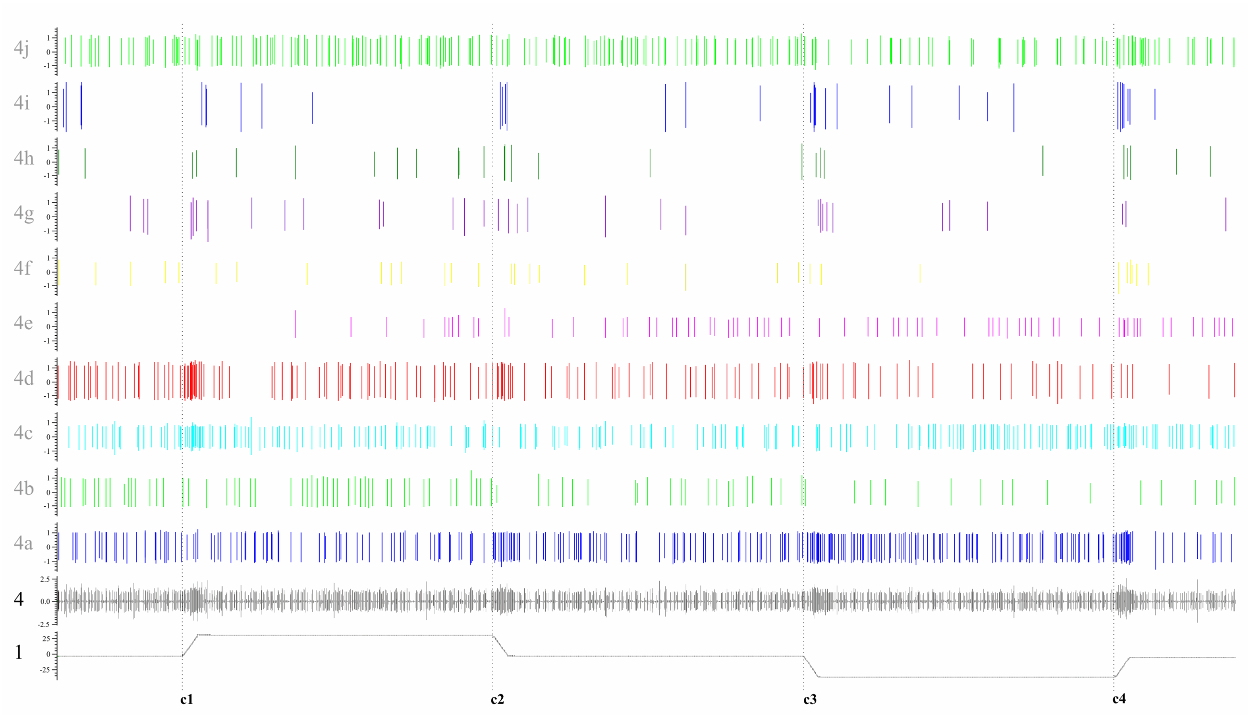

Fig. S10. Spike sorting reveals single units activity in *n. vestibularis* recording. 1 — Position marker; 4 — extracellular recording of *n. vestibularis*. 4a-4j – virtual channels (single units) derived from EC trace. Inter-stimulus interval is 60 s.
